# Epigenetic Reprogramming in *Mist1^−/−^* Mice Predicts the Molecular Response to Cerulein-Induced Pancreatitis

**DOI:** 10.1371/journal.pone.0084182

**Published:** 2014-01-21

**Authors:** Rashid Mehmood, Gabor Varga, Sonali Q. Mohanty, Scott W. Laing, Yuefeng Lu, Charis L. Johnson, Alexei Kharitonenkov, Christopher L. Pin

**Affiliations:** 1 Children's Health Research Institute, London, Ontario, Canada; 2 Children's Health Research Institute, Department of Paediatrics, London, Ontario, Canada; 3 Children's Health Research Institute, Department of Physiology & Pharmacology, London, Ontario, Canada; 4 Oncology, the University of Western Ontario, London, Ontario, Canada; 5 Lilly Research Laboratories, Eli Lilly and Company, Indianapolis, Indiana, United States of America; 6 Biostatistics and Programming, Sanofi US, Framingham, Massachusetts, United States of America; Centro Nacional de Investigaciones Oncológicas (CNIO), Spain

## Abstract

Gene expression is affected by modifications to histone core proteins within chromatin. Changes in these modifications, or epigenetic reprogramming, can dictate cell fate and promote susceptibility to disease. The goal of this study was to determine the extent of epigenetic reprogramming in response to chronic stress that occurs following ablation of MIST1 (*Mist1^−/−^*), which is repressed in pancreatic disease. Chromatin immunoprecipitation for trimethylation of lysine residue 4 on histone 3 (H3K4Me3) in purified acinar cells from wild type and *Mist1^−/−^* mice was followed by Next Generation sequencing (ChIP-seq) or ChIP-qPCR. H3K4Me3-enriched genes were assessed for expression by qRT-PCR in pancreatic tissue before and after induction of cerulein-induced pancreatitis. While most of H3K4Me3-enrichment is restricted to transcriptional start sites, >25% of enrichment sites are found within, downstream or between annotated genes. Less than 10% of these sites were altered in *Mist1^−/−^* acini, with most changes in H3K4Me3 enrichment not reflecting altered gene expression. Ingenuity Pathway Analysis of genes differentially-enriched for H3K4Me3 revealed an association with pancreatitis and pancreatic ductal adenocarcinoma in *Mist1^−/−^* tissue. Most of these genes were not differentially expressed but several were readily induced by acute experimental pancreatitis, with significantly increased expression in *Mist1^−/−^* tissue relative to wild type mice. We suggest that the chronic cell stress observed in the absence of MIST1 results in epigenetic reprogramming of genes involved in promoting pancreatitis to a poised state, thereby increasing the sensitivity to events that promote disease.

## Introduction

During development, coordinated transcription factor activity dictates the differentiation of multipotent pancreatic progenitor cells into exocrine and endocrine phenotypes [1]. This developmental transcription factor profile is re-activated during pancreatitis and pancreatic ductal adenocarcinoma [2], [3] suggesting de-dedifferentiation to a more progenitor-like state is an early event in disease progression [4]. There is a growing body of literature that indicates changes to the epigenome, or epigenetic reprogramming, dictate cell differentiation and development by promoting alterations in gene expression [5], [6], [7]. Epigenetic reprogramming involves post-translational modifications to either the DNA or histone core proteins within the chromatin [8] that often affect transcription factor binding. Methylation of cytosines is typically associated with gene repression, while histone acetylation correlates with transcriptional activity [8], [9]. Methylation of histone proteins can correlate with either active or repressed expression, depending on the amino acid targeted. Trimethylation of lysine 4 and 36 in histone 3 (H3K4Me3 and H3K36Me3) is generally associated with active genes [10], [11], while H3K9Me3 and H3K27Me3 are associated with silent genes [12], [13].

Studies during pancreatic development show dynamic changes in H3K4Me3 and H3K27Me3 enrichment at lineage specific genes as cells attain distinct differentiation properties [14], [15]. To date, global analysis of the adult pancreatic cell epigenome outside of pancreatic cancer has not been performed. Since epigenetic modifications that occur in response to changes in the environment lead to short or long-term consequences on gene expression [16], [17], having an epigenetic map of histone modifications is essential for understanding how the environment affects gene expression. Identifying epigenetic reprogramming in disease models also provides a basis for understanding the mechanisms by which adverse environmental events affect gene activity. In this study, we examined the global enrichment of H3K4Me3 in pancreatic acini from C57/Bl6 mice, the most common strain of laboratory mouse used, and in congenic mice carrying a targeted mutation of the *Mist1* gene.

MIST1 (or *Bhlha15*) is a basic helix-loop-helix (bHLH) transcription factor required for the differentiation of pancreatic acinar cells [18] and targets genes involved in cell communication (*Gjb1*, encodes Cx32;[19]) and exocytosis (*Rab3D* and *Rab26*;[20], [21]). However, *Mist1^−/−^* pancreatic tissue is under chronic stress [22] and many of the gene expression changes are likely not a direct consequence of MIST1's transcriptional activity. We have shown that the unfolded protein response is elevated in *Mist1^−/−^* pancreatic tissue [22], but is not further enhanced when these mice are exposed to additional acute stress [23]. *Mist1^−/−^* acinar cells exhibit many of the early stages of pancreatitis, including increased expression of pancreatitis-associated proteins, premature enzyme activation [18], and altered Ca^2+^ signalling [24]. In addition, *Mist1^−/−^* acini are significant more likely to undergo acinar-to-duct cell metaplasia relative to normal cells [25]. Therefore, *Mist1^−/−^* mice represent a unique animal model for understanding the relationship of chronic cell stress to epigenetic reprogramming that may promote increased susceptibility to pancreatic disease.

The goals of this study were to (a) define the global enrichment of H3K4Me3 in the pancreatic genome, (b) compare this pattern to what is observed in *Mist1^−/−^* pancreatic tissue, and (c) determine changes in H3K4Me3 enrichment that can be used as a guide for interpreting gene expression differences between the genotypes. We chose to focus on the H3K4Me3 since it is enriched at the transcription start site (TSS) and regulatory regions of active genes [11], [26], [27], [28]. H3K4Me3 enrichment has been studied in pancreatic development [14], [15], and H3K4Me3 enrichment sites are focused to less than 2 kb in breadth. Conversely, H3K27Me3 enrichment occurs over 50–100 kb, making it more difficult to identify affected genes [29].

Our results identify a reproducible epigenetic map for H3K4Me3 enrichment in pancreatic acinar cells including significant enrichment away from TSSs. While a number of regions were epigenetically reprogrammed for H3K4Me3 in *Mist1^−/−^* tissue, less than 20% of these changes correlated with altered gene expression. Ingenuity Pathway Analysis of genes differentially marked for H3K4Me3 between genotypes revealed a strong enrichment for genes involved in gastrointestinal diseases, pancreatitis and pancreatic ductal adenocarcinoma. Induction of acute pancreatitis in mice indicated that differential H3K4Me3 enrichment was at times predictive of gene activation, suggesting that *Mist1^−/−^* acini are primed for a more severe pancreatitis response. These findings have significant implications for the susceptibility to pancreatic disease and response to environmental factors that may promote disease.

## Materials and Methods

### Mice

All studies were conducted following animal protocol #2008-116, approved by the Animal Care and Use Subcommittee at the University of Western Ontario. Mice containing a deletion of *Mist1* (*Mist1^−/−^*) have been described [18] and both wild type and *Mist1^−/−^* mice were on a C57/Bl6 background. Cerulein-induced pancreatitis was performed as described, and mice sacrificed four hours after initial cerulein or saline (control) injections [23]. N values for each experiment are indicated in the figure legends. Two colonies of mice, in different breeding facilities, were used in this study.

### Acinar cell isolation, Chromatin Immunoprecipitation and Next Generation sequencing

For a complete description of the Chromatin Immunoprecipitation (ChIP) protocol please see (http://www.pancreapedia.org/tools/methods/chromatin-immunoprecipitation-chip-from-pancreatic-acinar-cells-and-whole-pancreatic-t; DOI: 10.3998/panc.2011.24). Sonicated chromatin was prepared from acinar cells isolated from pancreatic tissue of two-month old WT and *Mist1^−/−^* mice as described [25] or from whole pancreatic tissue as indicated. ChIP was performed on chromatin samples with antibodies specific for H3K4Me3 or H3K27Me3 (Millipore). H3K4Me3 ChIP was followed by Next Generation sequencing (ChIP-seq). For ChIP-seq experiments, chromatin was pooled from acini purified from two mice of each genotype. Enriched DNA fragments were sequenced using the Illumina 2.0 genome analyzer (Beijing Genomics Institute, Hong Kong). The sequenced data was mapped to the mm9 mouse genome using a SOAP 2.21 alignment program [30] and has GEO Accession #GSE49113. The MACS 1.4.0 algorithm was used for peak calling [31], which represents the epigenetic marks compared to input samples. See **Figure S1** for parameters used for peak and gene calling. Wiggle (.wig) files generated by MACS were aligned to the mm9 mouse genome using the University of California at Santa Cruz (UCSC) genome browser gateway to visually analyze H3K4Me3 peaks (http://genome.ucsc.edu/cgi-bin/hgGateway). The pathway enrichment and disease association analysis was generated through the use of Ingenuity Pathway Analysis (Ingenuity Systems, www.ingenuity.com). We also compared ChIP-seq results to previously published microarray [32] data accession GSE34232. To validate ChIP-seq data, ChIP-qPCR was performed on chromatin prepared from at least three animals for each genotype.

### RNA isolation and RT-PCR analysis

RNA from untreated, saline, or cerulein-treated mouse pancreatic tissue was isolated using TRIZOL reagent (Invitrogen, Burlington, ON). cDNA was synthesized using ImPromII Reverse transcriptase (Sigma, St. Louis, MO). PCR was carried out using GoTaq flexi DNA polymerase (Sigma) using gene specific primer sequences. All ChIP-qPCR and qRT-PCR was performed using the GoTaq PCR Mastermix system (Promega, Madison WI) and samples evaluated using the ViiA^Tm^7 Real Time PCR System and corresponding ViiA^Tm^7 software (Applied Biosystems, Foster City, CA). Average C_t_ values for individual ChIP and IgG controls were expressed as a percent of input. The sequences of primers used are shown in **Table S1**.

### Statistical Analysis

**Figure 1:**
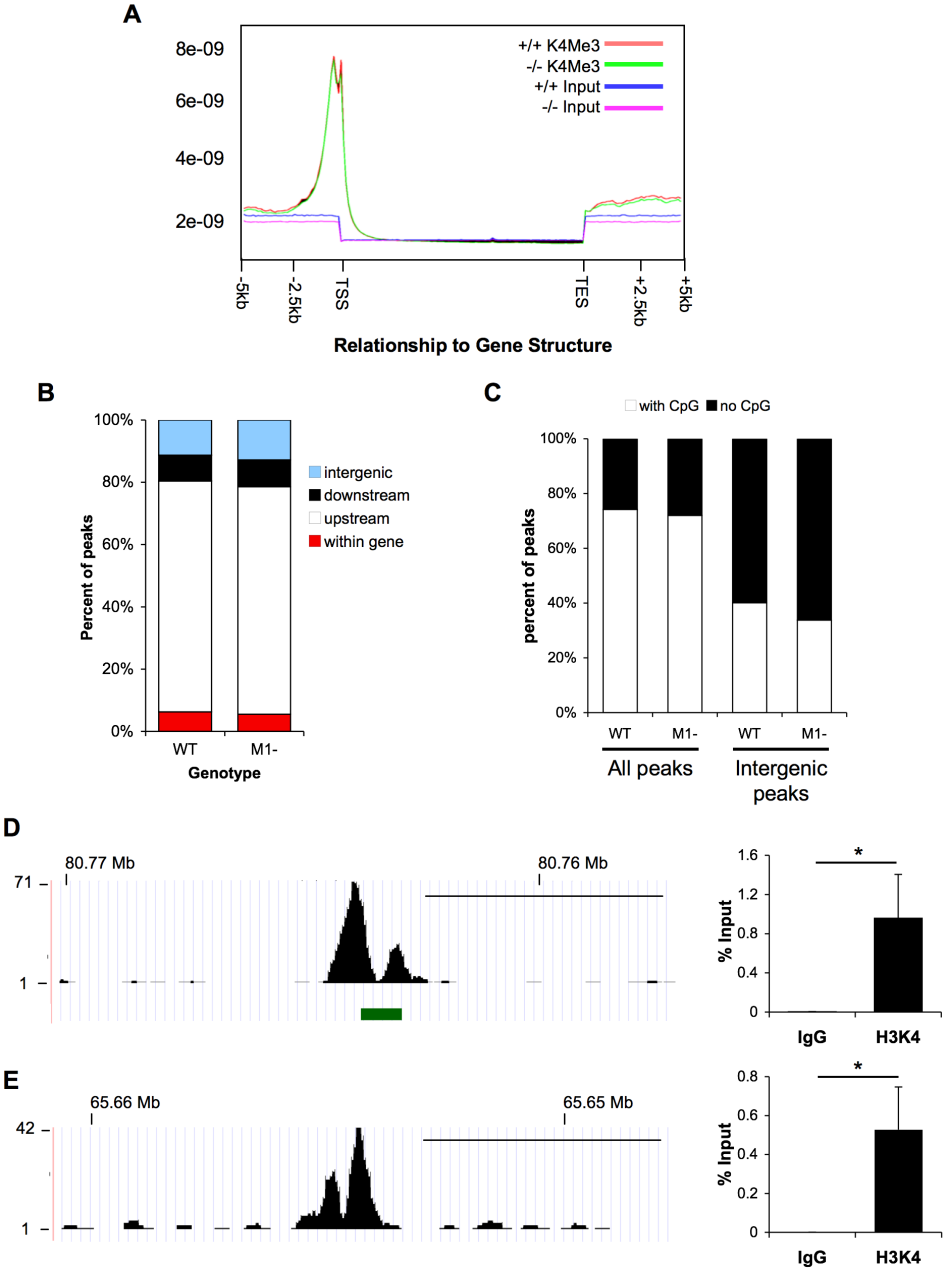
Global comparison of H3K4Me3 enrichment in pancreatic chromatin from wild type and *Mist1^−/−^* mice. **A**) Schematic analysis of global H3K4Me3 enrichment relative to transcriptional start sites (TSS) and transcriptional end sites (TES). Analysis was carried out for genomic regions spanning 5 kb upstream of the TSS or downstream of the TES. The majority of H3K4Me3 peaks concentrated to the TSS for wild type (+/+; red line) and *Mist1^−/−^* (−/−; green line). Analysis of input samples showed no peak enrichment through this region. Y-axis represents p-value of the specific region based on the unique mapped reads. The region would be defined as a peak when p-value<1×10^−5^. (**B**) Quantification of H3K4Me3 enrichment site in relation to specific genomic regions. Peaks classified as downstream and upstream are within 2 kb of TES or TSS, respectively. (**C**) Correlation of H3K4Me3 enrichment sites with CpG islands across the entire genome or related specifically to intergenic sites of enrichment. (**D, E**) Schematic representation of intergenic H3K4Me3 enrichment derived from ChIP-seq analysis on chromosome 7 with (**D**) or without (**E**) CpG islands. The green bar underneath the graphs depicts the location of a CpG island. Scale bar = 50 kb. In each case, the peaks were shown to be consistently present and significant through ChIP-qPCR were normalized to input levels (n = 4; *P<0.05). ChIP-qPCR for IgG was used as a control.

ChIP-qPCR and qRT-PCR were compared using either a Mann Whitney, two-tailed t test (**Figure 1**), or unpaired t tests. P values were determined using a Bonferroni post-hoc test. Values are provided with error bars representing the mean ± standard error.

## Results

To obtain a global perspective of H3K4Me3 enrichment after pancreatic differentiation, ChIP-seq was performed on pancreatic acinar cells isolated from two month-old male mice. ChIP-seq generated 12 million, 49-bp, raw reads. Following filtering to eliminate adaptor sequences, contamination, and low quality sequences, >90.6% of all sequences were mapped to the mm9 reference mouse genome (**Table 1**) with a unique mapped rate of 77.3%. This allowed for aligning >9.2 million unique sequences. Following alignment of the sequences to the annotated genome and MACS analysis to call peaks, the majority of H3K4Me3 enrichment (74.1%) localized to within two kilobases upstream (5′) of known transcriptional start sites (TSS; **Figure 1A, B**). We chose two kilobases as a cut off point since the range of H3K4Me3 peaks is less broad than other epigenetic modifications and known to be closely associated to TSSs [28], [33]. The alignment revealed that a significant proportion of H3K4Me3 marks are not associated with TSSs, located in intergenic regions (11.3%), within 2 kb of the transcriptional end site (TES; 8.3%) or within an annotated gene sequence (6.3%).

**Table 1:** Quality of ChIP-seq data.

	Wild Type		*Mist1^−/−^*	
Sequence data	H3K4Me3	Input	H3K4Me3	Input
**Raw Reads**	12,000,000	12,000,000	12,000,000	12,000,000
**Low Quality**	35624	60362	26598	64237
**Clean Data (rate)**	99.70%	99.50%	99.78%	99.46%
**Total Reads**	11,964,376	11,939,638	11,973,402	11,935,763
**Mapped Reads**	10,836,399	11,536,532	11,358,011	11,586,545
**Unique Mapped Reads**	9,244,322	9,098,237	9,591,198	9,416,926
**Mapped Rate**	90.57%	96.62%	94.86%	97.07%
**Unique Mapped Rate**	77.27%	76.20%	80.10%	78.90%

We defined intergenic peaks of H3K4Me3 enrichment to be localized at least 2 kb away from annotated gene sequence found in the Ensembl, NCBI and UCSC data bases. H3K4Me3 has association with both TSS and distal enhancer regions [34], so these intergenic peaks may represent undefined gene regulatory elements. In support of such a role, 74.1% of all H3K4Me3 enrichment sites were associated with CpG islands, which typically represent gene regulator sites (**Figure 1C**). However, only 40.1% of intergenic enrichment sites correlated to CpG islands (**Figure 1C**). To ensure that the intergenic peaks did not represent spurious sequencing from a single sample, we performed ChIP-qPCR on chromatin samples obtained from three additional mice housed in different breeding facilities for intergenic peaks with and without CpG islands on chromosome 7 (**Figure 1D, E**; respectively). In confirmation of the ChIP-seq data, H3K4Me3 enrichment was readily observed, indicating that these H3K4Me3 marks are consistent epigenetic modifications within the acinar cell genome. Based on the global analysis of H3K4Me3 enrichment in pancreatic acinar cells, we have developed an epigenetic road map for this epigenetic modification that provides novel information regarding acinar gene activity and suggests additional regulatory targets that H3K4Me3 may affect within the acinar cell genome.

To determine how the epigenetic program was affected in a model of pancreatic injury, we evaluated the H3K4Me3 program in *Mist1^−/−^* mice, which exhibit chronically elevated levels of pancreatic cellular stress [22], and are sensitive to factors promoting pancreatitis [23] and acinar-to-duct cell metaplasia [25]. Microarray analysis comparing WT and *Mist1^−/−^* pancreatic gene expression has been performed [32] and allows for correlation of H3K4Me3 enrichment and gene expression. The quality of the *Mist1^−/−^* ChIP-seq data was equivalent to the WT analysis (**Table 1**). 94.9% of the 12 million sequences could be aligned to the mm9 reference mouse genome with a unique mapped rate of 80.1%. In total, almost 9.6 million unique sequences were mapped to the mm9 genome. Peak calling revealed a slight decrease (∼2%) in the number of peaks in the *Mist1^−/−^* sample (14,936 peaks called) relative to the total number of H3K4Me3 peaks (15,233) identified in wild type tissue (**Figure S1**).

ChIP-seq data revealed similar global trends in the two genotypes (**Figure 1A, B**). The majority of H3K4Me3 peaks in the *Mist1^−/−^* pancreas (73.1%) were located within 2 kb upstream of known TSS (**Figure 1B**) with no global differences observed in the percentage of peaks aligning to intergenic or intragenic regions relative to TSSs (**Figure 1B**). The distribution (**Figure S2**) and number (**Figure S3**) of H3K4Me3 enriched areas across each chromosome within the *Mist1^−/−^* pancreas was similar to the distribution observed in WT tissue, as was the association of H3K4Me3 enrichment to CpG islands in *Mist1^−/−^* chromatin across the entire genome or intergenic regions (**Figure 1C**). Therefore, the loss of MIST1, and the phenotypic abnormalities resulting from this loss, does not lead to widespread changes in H3K4Me3 enrichment. Instead, epigenetic changes appear to be targeted.

**Figure 2:**
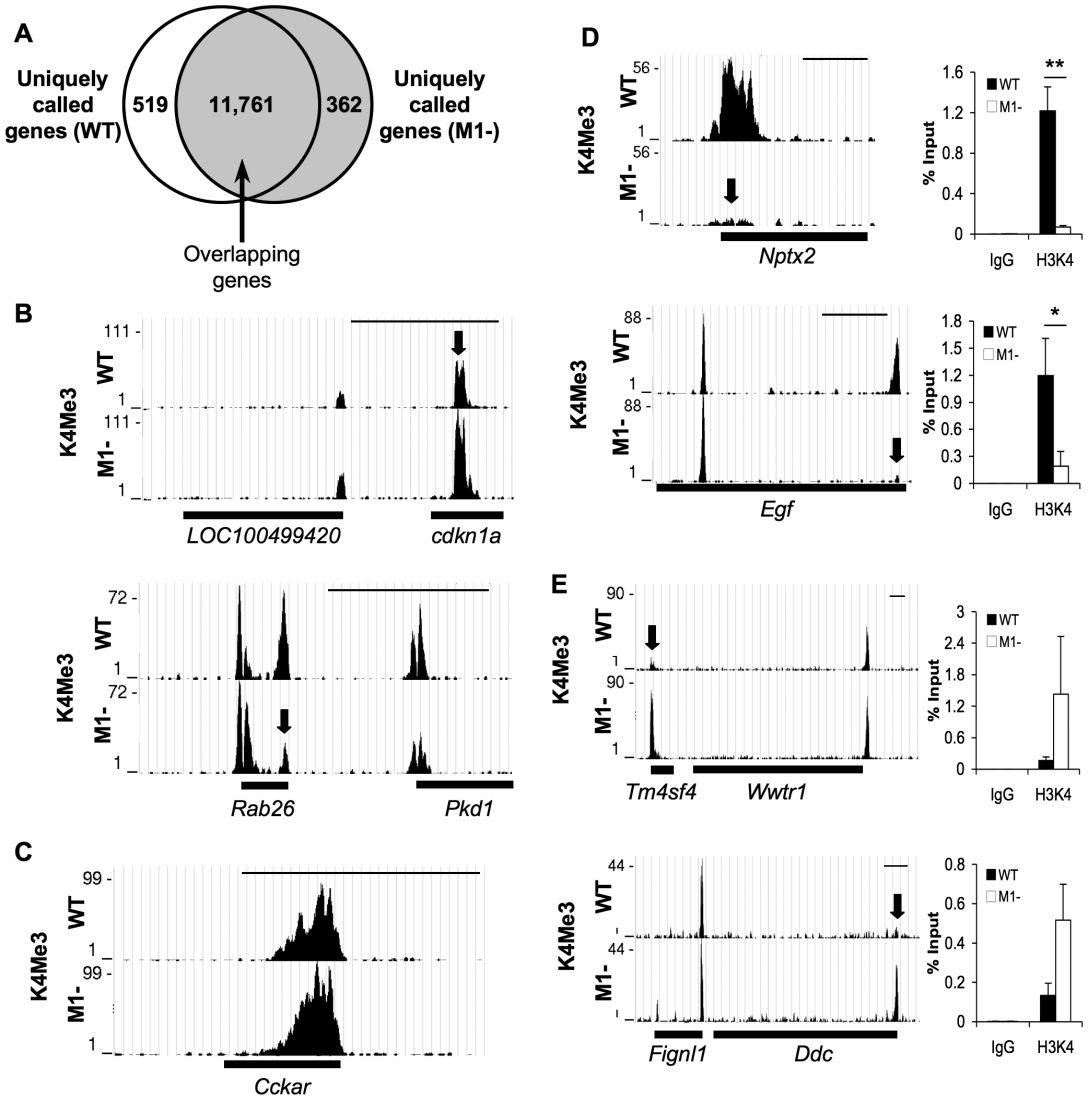
Differences in H3K4Me3 enrichment between WT and *Mist1^−/−^* pancreatic tissue are targeted. (**A**) Overlap of called genes identifies subsets of genes that are preferentially enriched for H3K4Me3 in WT or *Mist1^−/−^* tissue. (**B, C**) Schematic representation of ChIP-seq data showing H3K4Me3 enrichment for genes previously published with (**B**) decreased expression (*Cdkn1a*, *Rab26*) or (**C**) increased expression (*Cckar*) in *Mist1^−/−^* (M1-) pancreatic tissue. (**D, C**) Schematic representation of ChIP-seq data and validation by ChIP-qPCR showing H3K4Me3 enrichment for genes enriched for H3K4Me3 in (**D**) WT (*Nptx2, Egf*) or (**E**) *Mist1^−/−^* tissue (*Tm4sf4, Ddc*). Arrows indicate peak differences. Bars under the graph depict the extent of the genes. Scale bar = 50 kb (**B, C**) or 10 kb (**D, E**). Graphs represent ChIP-qPCR data verifying the differences in H3K4Me3 enrichment for each gene (n = 3). ChIP-qPCR was normalized to input levels. *P<0.05; **p<0.01.

To identify genes that were enriched for H3K4Me3, genes were “called” if a peak fell within one kilobase of a TSS. This resulted in 12,280 H3K4Me3-enriched genes within WT pancreas, and 12,123 H3K4Me3-enriched genes within the *Mist1^−/−^* pancreas, a difference of 1.3% (**Figure S1**). In total, there were 884 differences in the H3K4Me3 gene enrichment between these two samples. 519 genes were uniquely enriched only in WT tissue (4.2%), while 362 genes (3%) were enriched only in *Mist1^−/−^* tissue (**Figure 2A** **; Table S2**). The obvious explanation for the changes in H3K4Me3 enrichment between WT and *Mist1^−/−^* tissue is that genes associated with epigenetic changes are targets of MIST1 transcriptional activity. Surprisingly, none of the genes previously identified as targets of MIST1 activity in *Mist1^−/−^* pancreatic tissue appeared on the list of called genes. Visual scans of the ChIP-seq data confirmed similar peaks for H3K4Me3 enrichment in WT and *Mist1^−/−^* tissue for *Cdkn1a* and *Itpr3* (**Figures 2B** **,S4A**), which are expressed to lower levels in *Mist1^−/−^* mice [18], [35], or *Cckar*, *Prkce* and *Nupr1* (**Figures 2C** **,S4B**), which are expressed to higher levels in *Mist1^−/−^* mice [18], [25]. However, the visual scan also showed a reduction in the amplitude of H3K4Me3 enrichment for *Rab26*, *Rab3d* and *Gjb1* (**Figures 2B** **, S4B**), which are differentially expressed in the *Mist1^−/−^* pancreas [20], [21]. The trend in differential enrichment of *Gjb1* and *Rab26* for H3K4Me3 was likely not identified due to the rigorous statistical criteria applied by the MACS analysis, where only those peaks showing a complete absence of H3K4Me3 in either WT or *Mist1^−/−^* tissue were called.

**Table 2:** Differentially enriched genes related to pancreatitis through Ingenuity Pathway Analysis.

Gene Name	Evidence	PRO	ANT	References^1^	*p-value
***Genes Preferentially Enriched for H3K4M3 in Mist1^−/−^ pancreatic tissue***					
*Pnliprp1*	⇓expression in autoimmune pancreatitis relative to CP	X		[1]	0.016
*Gc*	⇓expression in autoimmune pancreatitis		X	[2]	1.1×10^−8^
*Ripk3*	⇓susceptibility for pancreatitis in *Rip3k^−/−^* mice	X		[3]	0.819
*Icam1*	activated early in experimental pancreatitis	X		[4]	0.703
*Gucy1b3*	clinical trials using activators to reduce systemic complications of ECRP-induced pancreatitis		v		.216
*Cd38*	expressed in neutrophils in AP	X		[5]	.991
*Cftr*	mutant *Cftr* associated with HP	X			.985
*Ptgs2 (Cox2)*	⇓susceptibility for pancreatitis in *Ptg2^−/−^* mice	X		[6]	.623
*Chrm1*	blocking muscarinic signaling abolishes pathology in alcohol induced pancreatitis		v	[7]	.374
***Genes Preferentially Enriched for H3K4M3 in wild type pancreatic tissue***					
*E2f1*	*E2f1^−/−^* mice develop pancreatic insufficiency and diabetes		X	[8]	0.009
*Mme*	inhibition ⇑ severity of pancreatitis		X	[9]	0.165
*Pde7b*	Inhibitors used to ⇓ systemic complications of ECRP–induced pancreatitis	v			0.688
***Genes Preferentially Enriched for H3K4M3 in Mist1^−/−^ pancreatic tissue***					
*Cftr*	mutations increase risk of PDAC				0.985
*Ptgs2*	⇑development of PDAC	X		[10]	0.623
*Serpine2*	promotes invasion of PDAC cells	X		[11], [12]	0.181
*Rara*	⇑in PDAC/expressed in PDAC cell lines	X		[13]	0.002
*Palld*	promotes invasion of pancreatic cancer	X		[14]	0.116
*Timp1*	induces hyperproliferation of PDAC cells	X		[15]	0.285
*mir-125b1*					N.D.
*Brsk2*	⇑ in PDAC	X		[16]	0.272
*Hhip*	⇑ in PDAC	X		[17]	0.738
*Pik3cg*	⇑ in PDAC; potential “driver mutation”	X		[18], [19]	0.143
*Flt4*	⇑ in PDAC	X		[20]	0.978
*mir-210*	⇑ increased expression in PDAC	X		[21]	N.D.
***Genes Preferentially Enriched for H3K4M3 in wild type pancreatic tissue***					
*Hoxb2*	⇑ upregulated in PDAC	X		[22]	0.162
*Tll1*	silenced in PDAC		X	[23]	0.336
*Pdgfra*	inhibition of PDGF signaling reduced human cancer growth and metastasis	v		[24]	0.285
*Rrm2*	⇑chemoresistance to gemcitabine	X		[25]	0.001
*Loxl2*	Inhibition reduces resistance to gemcitabine	X		[26]	0.32
*Vim*	⇑ in PDAC	X		[27]	0.411
*Plau*	⇑ expression associated with pancreatic cancer	X		[28]	0.797
*mir125b1*					N.D
*Egf*	Signaling essential for K-ras oncogene driven PDAC	X		[29]	2.42×10^−9^
*Krt7*	⇑ expression associated with pancreatic cancer	X		[30]	0.058
*Id1*	*Id* knockdown inhibits metastasis	X		[31]	0.641
*Nptx2*	silenced in PDAC; inhibits proliferation and metastasis		X	[32], [33]	0.606
*Axl*	⇑ expression promotes invasion/survival of PDAC	X		[34]	0.206
*Foxa1*	downregulation required for EMT		X	[35]	0.578
*Anxa1*	suppresses NFkB		X	[36]	0.580
*Rasgrf2*	methylation observed in PDAC		X	[23]	0.468

PRO – promotes pancreatitis; ANT – reduces severity of pancreatitis; X - clear association; v – weak association;

* obtained from DiRenzo et al (2012) – p<0.05 was used as a cut-off for differentially expressed.

1 - see supplemental information for references.

Therefore, we decided to expand out criteria for changes in H3K4Me3 enrichment to include genes that were *preferentially* enriched for H3K4Me3. By inputting the .wig files for each chromosome into the UCSC genome browser, the entire ChIP-seq data was visually scanned to identify sites that showed at least a two-fold difference in the amplitude of H3K4Me3 enrichment between genotypes. To be included, the H3K4Me3 peak needed to have an amplitude of at least 10 and to be within 1 kb of an annotated gene sequence. This visual analysis expanded the list of called genes from 519 unique genes to 769 genes (6.3% of the total number of called genes) that are preferentially H3K4Me3-enriched in WT pancreatic tissue, and from 362 unique genes to 625 preferentially H3K4Me3-enriched genes (5.2%) in *Mist1^−/−^* mice (**Table S2).**

To validate differences in H3K4Me3 enrichment, targeted ChIP-qPCR analysis was performed on pancreatic chromatin for three separate mice. We chose genes that showed unique (*Nptx2*) or preferential (*Egf*) enrichment for H3K4Me3 in WT tissue (**Figure 2D**). ChIP-qPCR confirmed increased H3K4Me3 enrichment on both genes supporting the ChIP-seq data. Conversely, *Tm4sf4* and *Ddc* showed increased enrichment for H3K4Me3 in *Mist1^−/−^* tissue based on ChIP-qPCR (**Figure 2E**). These results show that the differences in H3K4Me3 enrichment identified by ChIP were consistent in all *Mist1^−/−^* pancreatic samples and represented targeted changes in H3K4Me3 enrichment between the genotypes.

**Figure 3:**
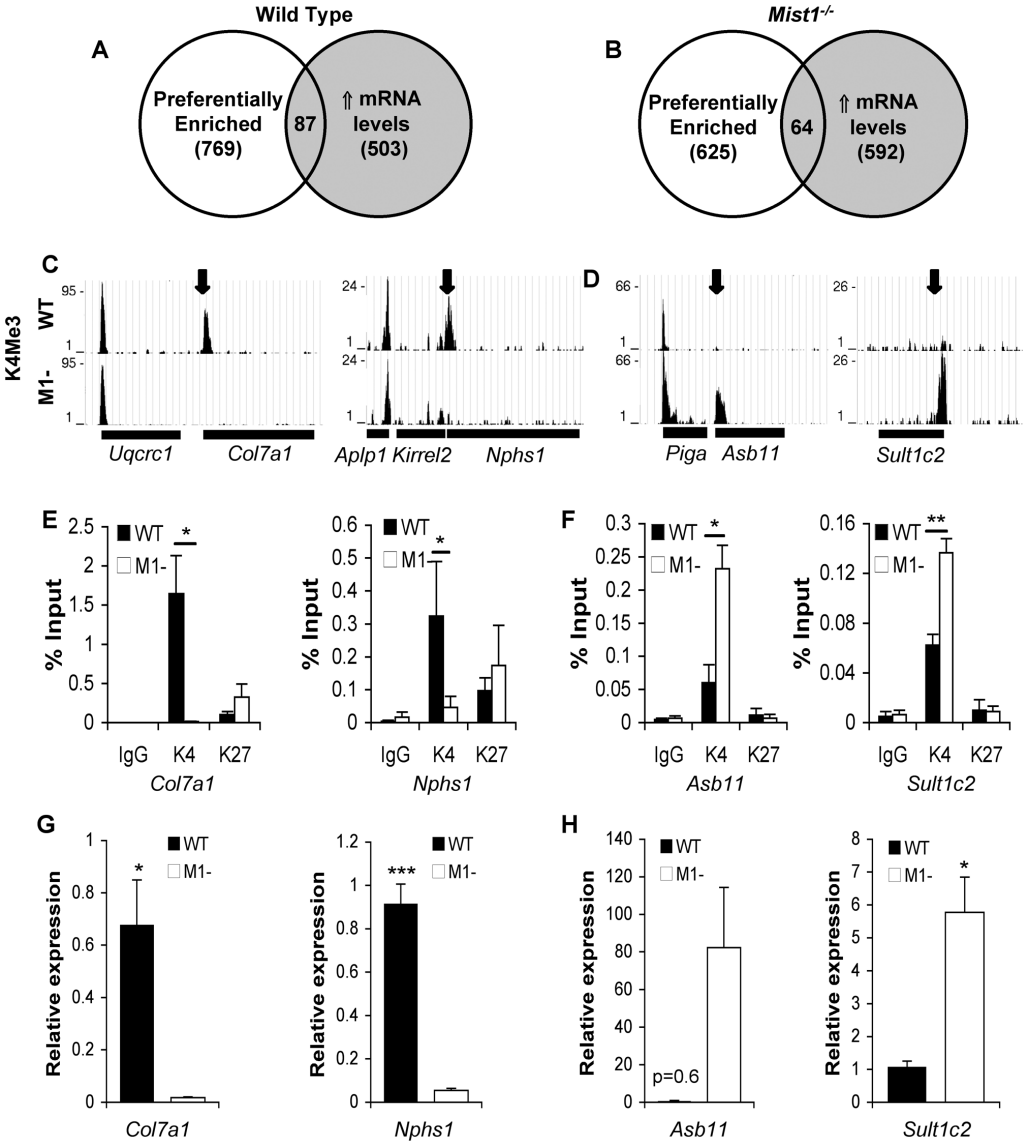
A subset of genes that are differentially enriched © WT and *Mist1^−/−^* pancreatic tissue exhibit altered gene expression. (**A, B**) Overlap of data sets for genes that are preferentially enriched for H3K4Me3 and have increased expression based on microarray analysis (DiRenzo et al, 2012; GEO Accession # GSE34232) in (**A**) wild type and (**B**) *Mist1^−/−^* tissue. Schematic representation of ChIP-seq data showing H3K4Me3 enrichment for genes uniquely enriched for H3K4Me3 in (**C**) WT or (**D**) *Mist1^−/−^* tissue. Bars under the graph depict the extent of the genes. (**E, F**) ChIP-qPCR for H3K4me3 and H3K27Me3 enrichment, and (**G, H**) qRT-PCR for expression of genes identified by ChIP-seq. ChIP-qPCR was normalized to input levels *P<0.05; **P<0.01; ***P<0.001; n = 3.

To determine if changes in H3K4Me3 enrichment between genotypes is indicative of changes in gene expression, we compared the results of the ChIP-seq data with a recent microarray analyses [32] contrasting pancreatic gene expression between two-month old wild type and *Mist1^−/−^* mice. To be identified as differentially expressed, we chose those genes exhibiting at least 1.5-fold difference in expression with a P value<0.01. Using these criteria, 503 genes were identified with increased expression in WT tissue and 592 genes with increased expression in *Mist1^−/−^* pancreas. Of the 503 genes with increased expression in WT tissue, only 26 (5.2%) were uniquely enriched for H3K4Me3 in WT pancreas. Expanding this comparison to include preferentially enriched genes identified 87 of the 503 genes (17.3%; **Figure 3A**). Similar analysis revealed 31 of 592 differentially expressed genes (5.2%) with unique H3K4Me3 enrichment, or 64 of 592 genes with preferential enrichment (10.8%) in *Mist1^−/−^* pancreas (**Figure 3B**). The complete comparison is provided in **Table S2**. We confirmed differential enrichment of H3K4Me3 and gene expression for a subset of genes identified in both lists by ChIP-qPCR, and qRT-PCR, respectively. In each case, ChIP-qPCR results confirmed the ChIP-seq data (**Figure 3C–F**). As indicated by the ChIP-seq data, greater H3K4Me3 enrichment was observed in WT chromatin for *Col7a1* and *Nphs1*, while *Asb11* and *Sult1c2* showed increased H3K4Me3 enrichment in *Mist1^−/−^* samples. A direct correlation between H3K4Me3 enrichment and gene expression was confirmed by qRT-PCR (**Figure 3G, H**). To determine if changes in gene expression may be correlated to epigenetic silencing, ChIP-PCR was performed for H3K27Me3. No changes in H3K27Me3 enrichment were observed at these genes. The limited correlation between differences in gene expression and H3K4Me3 enrichment was surprising and suggests that changes in H3K4Me3, while targeted, are not indicative of altered expression.

**Figure 4:**
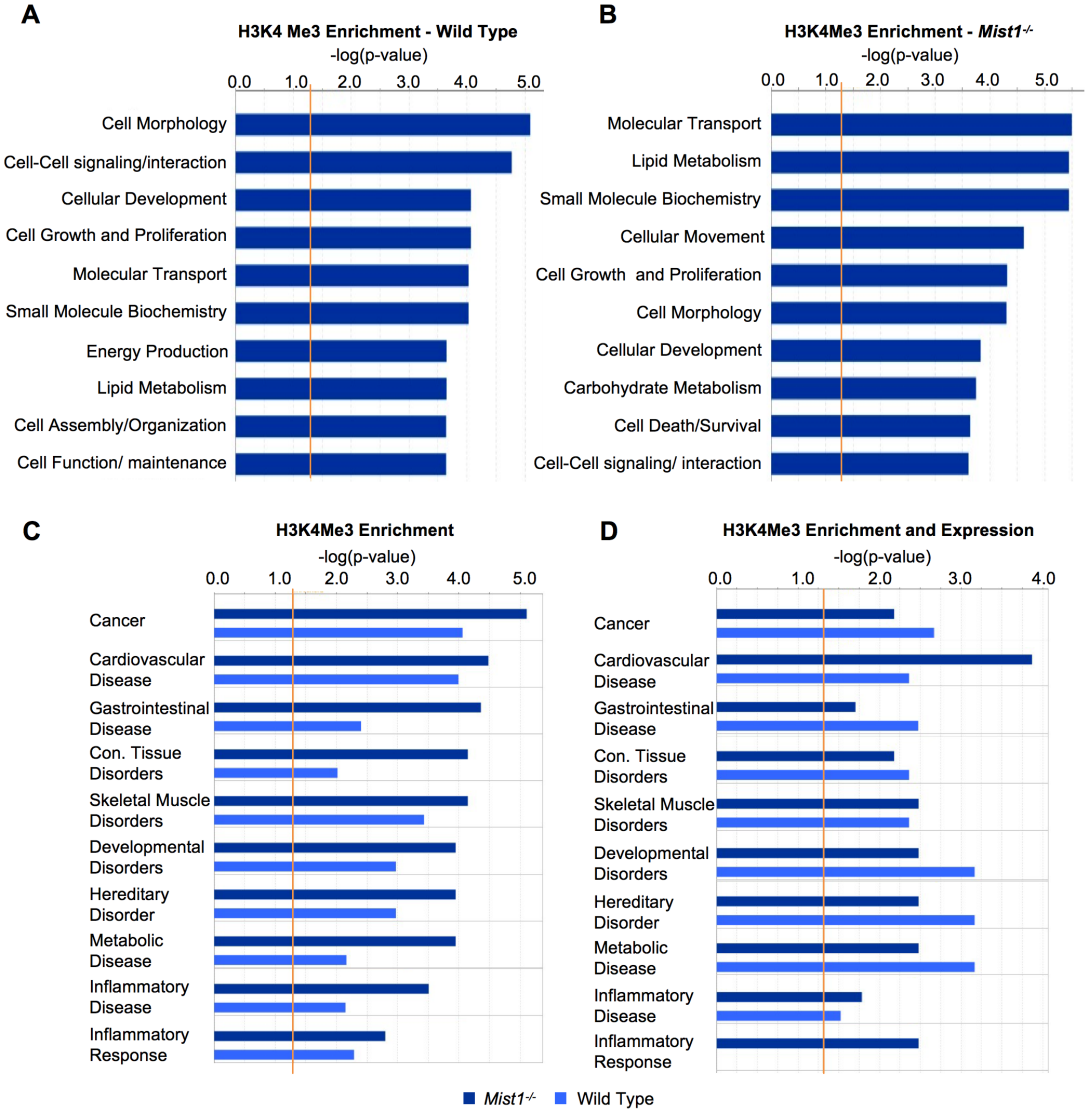
Specific pathways are selectively enriched between WT and *Mist1^−/−^* acinar cells. Ingenuity Pathway analysis (IPA) was performed for genes exhibiting preferential H3K4Me3 enrichment in either genotype. A right tailed Fisher's exact test was used to prioritize the most significant biological functions based on −log of p-value. The ratio was calculated by taking the number of genes in a given pathway that meet the cutoff criteria, and dividing this total by the number of genes that make up the pathway. The orange line represents the threshold where pathways are considered over-represented. The ten most represented molecular and cell _erulean_g pathways are shown for gene sets that are preferentially enriched for H3K4Me3 in (**A**) wild type and (**B**) *Mist1^−/−^* samples. (**C**) The top eight disease-related pathways represented by enriched genes within *Mist1^−/−^* samples are shown along with inflammatory disease and response, due to the relationship of MIST1 with pancreatitis. (**D**) Identical IPA performed for these disease related pathways using the list of genes that are differentially enriched and differentially expressed between WT and *Mist1^−/−^* tissue.

Since alterations in H3K4Me3 enrichment between genotypes is targeted, this suggests that the epigenetic changes may define specific biological processes affected by the loss of MIST1. Ingenuity Pathway Analysis (IPA) revealed the molecular pathways highly represented by differential gene enrichment in WT or *Mist1^−/−^* pancreatic tissue (**Figure 4A,B**). Interestingly, many pathways are identified as significantly represented in both genotypes. However, IPA only reflects an association with the pathway and does not indicate a positive or negative effect of the gene on that pathway. As would be expected from previous studies on the *Mist1^−/−^* phenotype, genes involved in cell morphology and cell-cell signalling are 10× more likely to be represented in WT samples, while genes involved in cell death and survival are 100× more likely to be represented in *Mist1^−/−^* tissue. Interestingly, there is a high representation for H3K4Me3-enriched genes involved in lipid metabolism in *Mist1^−/−^* tissue. An effect on lipid metabolism has not yet been identified as altered in *Mist1^−/−^* mice, and may reflect a possible metabolic defect in these mice.

A similar pathway analysis examining disease-related pathways identified increased representation for genes involved in cancer, gastrointestinal disease and connective tissue disorders in *Mist1^−/−^* samples (**Figure 4C**). This suggests pathways linked to these diseases might be preferentially activated within *Mist1^−/−^* tissue. However, pathway analysis for genes that exhibited alterations in both expression and H3K4Me3 enrichment showed no difference in the representation of these pathways between genotypes (**Figure 4D**). For example, genes involved in gastrointestinal disease were 100-fold more likely to be enriched in *Mist1^−/−^* tissue compared to WT tissue (**Figure 4C**), but less likely to expressed (**Figure 4D**). Therefore, gene expression was not altered in the same fashion as the epigenetic program in *Mist1^−/−^* mice.

**Figure 5:**
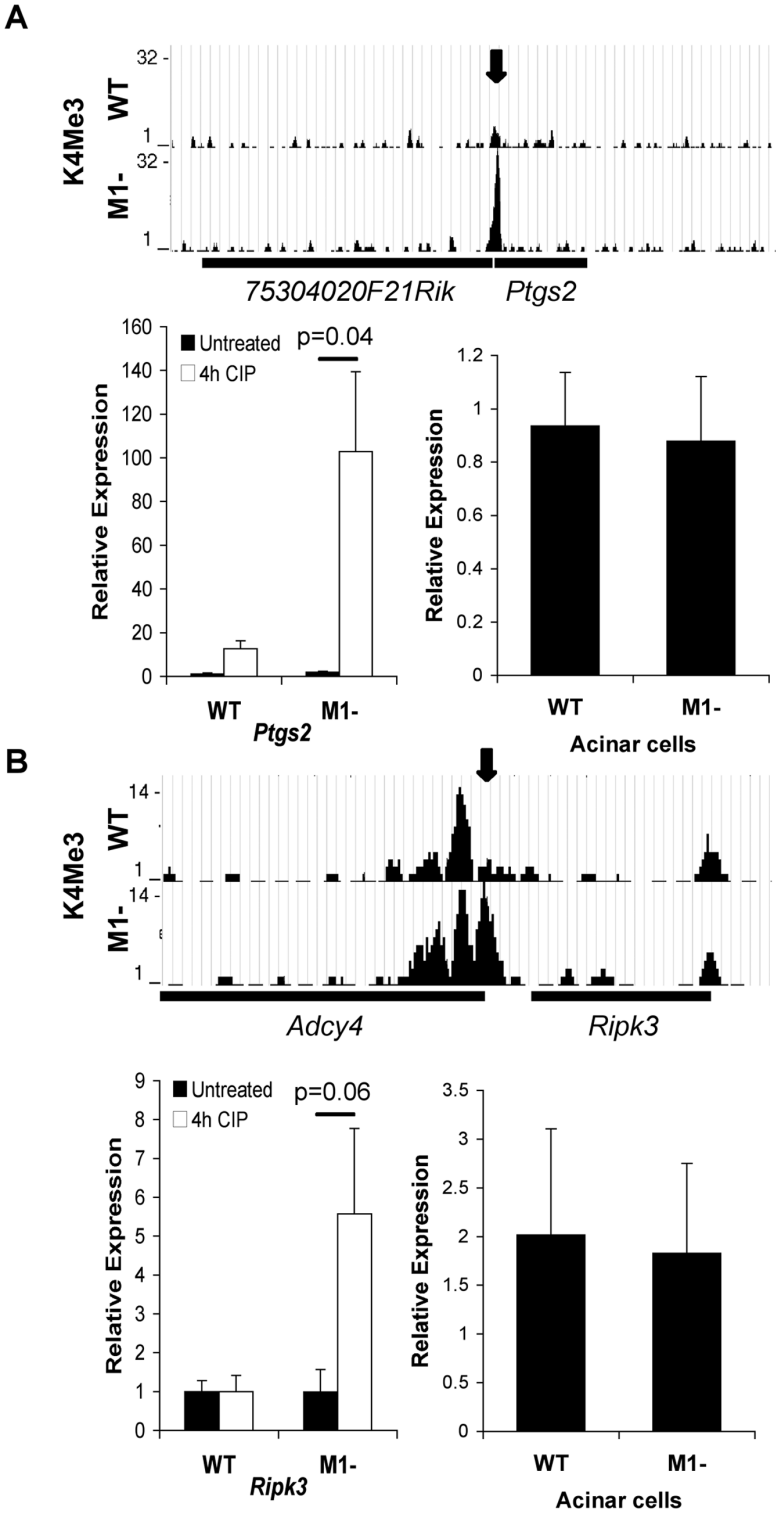
Epigenetic changes predict molecular responses to _erulean induced pancreatitis (CIP). Schematic representation of ChIP-seq data showing H3K4Me3 enrichment for (**A**) *Ptgs2* and (**B**) *Rpik3*, which were uniquely enriched for H3K4Me3 in *Mist1^−/−^* tissue but exhibit no change in gene expression. Arrows indicate peak differences. Bars under the graph depict the extent of the genes. Graphs represent qRT-PCR on whole pancreatic tissue from untreated or four hours of _erulean treatment in wild type (WT) and *Mist1^−/−^* (M1-) mice (left; n = 3), or immediately after acinar cell isolation (n = 4; right).

**Figure 6:**
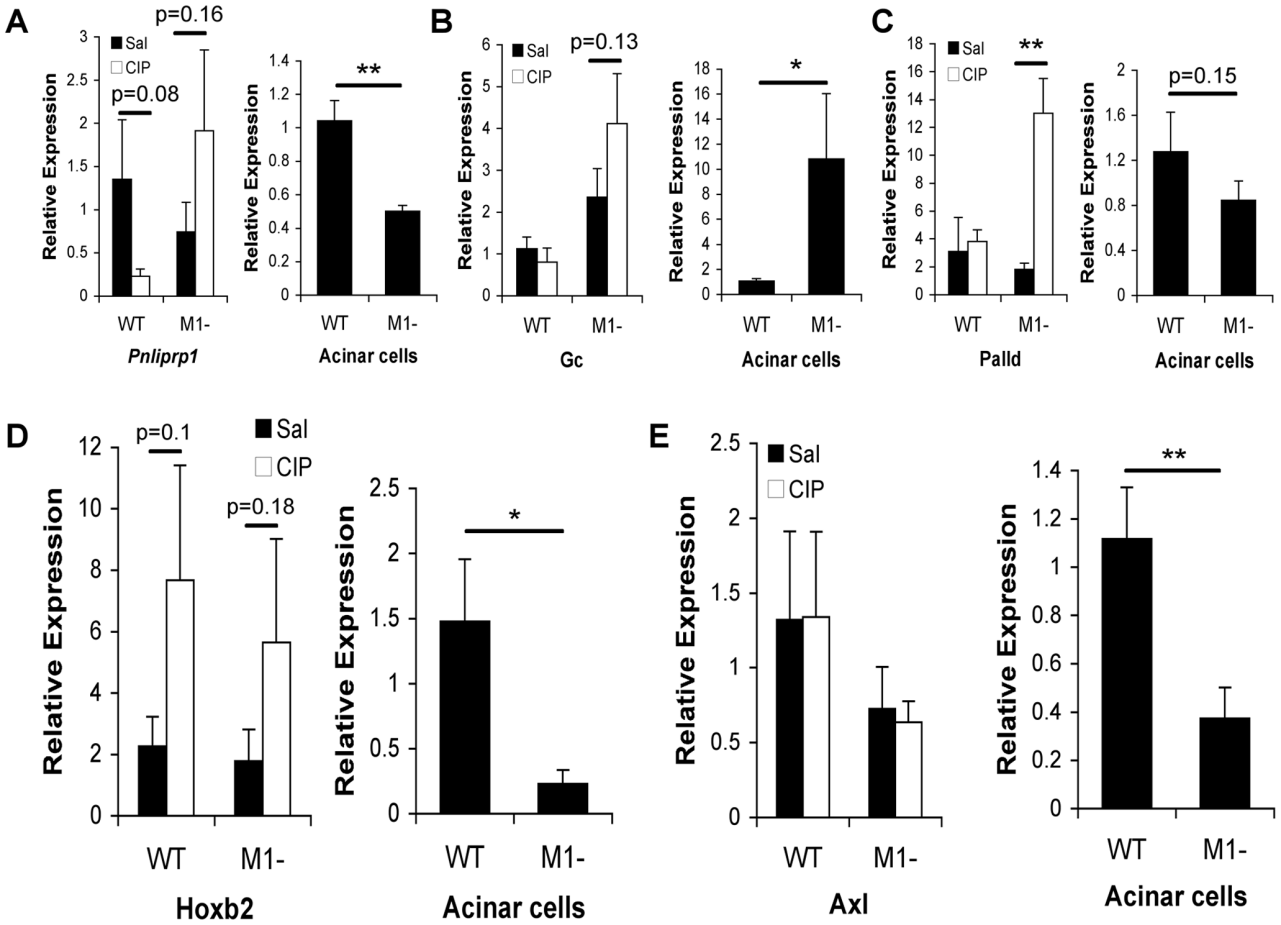
Differential response to _erulean treatment or acinar cell isolation. Gene expression analysis between (**A**) *Pnliprp1*, (**B**) *Gc*, (**C**) *Palld*, (**D**) *Hoxb2* and (**E**) *Axl*. Graphs represent qRT-PCR on whole pancreatic tissue 4 hours after saline or _erulean treatment of wild type (WT) and *Mist1^−/−^* (M1-) mice (left; n = 4), or immediately after acinar cell isolation (n = 4; right). *p<0.05; **p<0.01.

Since MIST1 is lost in pancreatitis and PDAC [25], we performed pathway analysis for genes associated to either disease. Genes linked to pancreatitis and exhibiting preferential H3K4Me3 enrichment in one genotype were more highly represented in *Mist1^−/−^* tissue (**Table 2A**). Interestingly, genes that promote pancreatitis were preferentially enriched for H3K4Me3 in *Mist1^−/−^* tissue, while genes correlating to decreased severity of pancreatitis were preferentially enriched in WT samples. While not as distinct, a similar epigenetic reprogramming was observed for genes involved in PDAC. 28 genes previously linked to PDAC were differentially enriched between the two genotypes (**Table 2B**). Surprisingly, only three genes identified for either disease (*Gc*, *Rrm2* and *Egf*) were also differentially expressed between WT and *Mist1^−/−^* mice based on microarray analysis. The differential enrichment between genotypes suggests that the epigenome may be primed to increase susceptibility for pancreatitis or PDAC in *Mist1^−/−^* mice. To assess this possibility, acute cerulein-induced pancreatitis was induced in WT and *Mist1^−/−^* mice and gene expression assessed 4 hours later. *Ripk3*, *Ptgs2*, and *Pnliprp1*, all preferentially enriched in *Mist1^−/−^* tissue, showed increased expression in *Mist1^−/−^* mice only after initiation of CIP (**Figures 5A, B** **,** **6A**). In fact, *Pnliprp1* expression appears to decrease in CIP-treated WT tissue. No difference in expression was observed between genotypes prior to initiating CIP or in purified acinar cells, although surprisingly, there was a trend towards decreased expression for *Pnliprp1* in *Mist1^−/−^* acinar cells relative to WT acini (**Figure 6A**). Gc, which was both differentially expressed and enriched for H3K4Me3, showed a trend for increased expression in saline-treated mice that was further exacerbated in culture (**Figure 6B**). We next examined *Palld*, a gene that has been strongly linked to familial pancreatic cancer [36], [37] and was differentially enriched for H3K4Me3 in *Mist1^−/−^* samples. Similar to *Ptgs2* and *Ripk3*, there was no difference in expression in saline-treated tissue or acinar cells. However, there was a dramatic increase in expression following CIP treatment only in *Mist1^−/−^* samples (**Figure 6C**). Finally, we examined two genes that were differentially enriched for H3K4Me3 only in WT mice, *Hoxb2* and *Axl*, and linked to pancreatic cancer. While Hoxb2 expression increased during CIP (**Figure 6D**), similar increases were observed in both genotypes. Alternatively, *Axl* showed no difference between genotypes or following CIP treatment (**Figure 6E**). Interestingly, both genes showed higher expression in WT acinar cells compared to *Mist1^−/−^* cells. This suggests that differential activation of gene expression is context dependent - not all H3K4Me3-enriched genes will be activated by 4 hours of cerulein treatment. Therefore, it appears that epigenetic changes related to genes involved in disease occur prior to changes in gene expression.

## Discussion

While the roles of specific transcription factors are well defined in the context of pancreatic development and disease, the involvement of epigenetic reprogramming is still undefined. In this study, we examined global H3K4Me3 enrichment in adult pancreatic tissue under normal and chronically stressed (*Mist1^−/−^*) conditions and developed an epigenetic road map for H3K4Me3 enrichment within the acinar cell genome. We showed that alterations in H3K4Me3 enrichment that occur under chronic stress conditions are targeted, and often these changes in H3K4Me3 do not reflect changes in gene expression. However, our results indicate that epigenetic reprogramming may lead to priming of genes, allowing for rapid induction in response to external cues. Such reprogramming provides a link between chronic stress and disease susceptibility and suggests changes in the epigenetic landscape of acinar cells are early events in promoting increased susceptibility to disease.

Global approaches for assessing gene regulation are highly insightful. Approaches such as gene microarray and RNA-seq have identified novel contributors to disease or genetic susceptibility, while ChIP-seq experiments have defined new gene targets for well-known transcription factors. However, ChIP-seq has the advantage of identifying microRNAs, lncRNAs and intergenic regulatory regions with specific epigenetic marks that are not usually covered by conventional ChIP-on-chip analysis. Considering the evidence that most of the mouse genome is transcribed [38], [39], [40], the ChIP-seq analysis for the unannotated regions of chromosomes becomes even more important. H3K4Me3 has been identified as an epigenetic modification associated with TSSs of active genes. We show that a significant proportion of H3K4Me3 sites (25.9%) is not associated with TSSs, located between or within genes, distal from the TSS. These peaks are not randomly located throughout the genome, but were consistently enriched in chromatin samples from multiple mice in different breeding facilities. Recent evidence suggests H3K4Me3 can be enriched at regulatory regions distal to TSSs [34]. Therefore, the presence of H3K4Me3 enrichment in these regions may define unknown, distal regulatory elements of active genes. Chromosome confirmation capture (3C) is necessary to align intergenic H3K4Me3 enrichment points with distally affected genes.

Numerous reports show that stress caused by environmental toxins or nutrition deficiency leads to a shift in epigenome [41], [42], leading us to predict that *Mist1^−/−^* pancreatic tissue would also be epigenetically reprogrammed. ChIP-seq analysis revealed targeted H3K4Me3 changes in *Mist1^−/−^* mice suggesting that specific molecular pathways are affected. Since MIST1 is a transcription factor, it could be suggested that reprogramming of H3K4Me3 simply reflects direct targets of MIST1 transcriptional activity. MIST1 is believed to enhance the expression of genes within the exocytosis pathway and the ChIP-seq analysis identified decreased H3K4Me3 enrichment for *Rab26*, *Rab3d* and *Gjb1*, direct targets of MIST1 [20], [21]. However, changes in H3K4me3 enrichment were not limited to genes regulated by MIST1 and alone were not sufficient to predict the gene expression changes between WT and *Mist1^−/−^* mice. In fact, the majority of gene expression changes between genotypes were not reflected by changes in H3K4Me3 enrichment. It is possible that the inconsistency between H3K4Me3 enrichment and gene expression may reflect the sources of material. Previous microarray have been performed on pancreatic tissue, while we used isolated acinar cells for ChIP-seq experiments. We and others have shown that acinar cell isolation can affect gene expression [25], [43], [44]. However, H3K4Me3 enrichment in the ChIP-seq samples has been validated for all genes examined by ChIP-qPCR (11 shown in this study). While we did observe gene expression difference between WT and *Mist1^−/−^* acini for *Hoxb2* and *Axl*, *Ptgs2*, *Ripk3*, *Palld*, and *Pnliprp1* all showed difference only during CIP treatment and not as a result of the isolation procedure. Therefore, while it is possible that H3K4Me3 enrichment could occur within the time frame of acinar cell isolation, our results still confirm that a subset of genes are differentially primed for activation during CIP. The observation that H3K4Me3 enrichment does not alter gene expression hints that epigenetic reprogramming is an initiating event that dictates the sensitivity of the pancreas to factors promoting disease.

**Figure 7:**
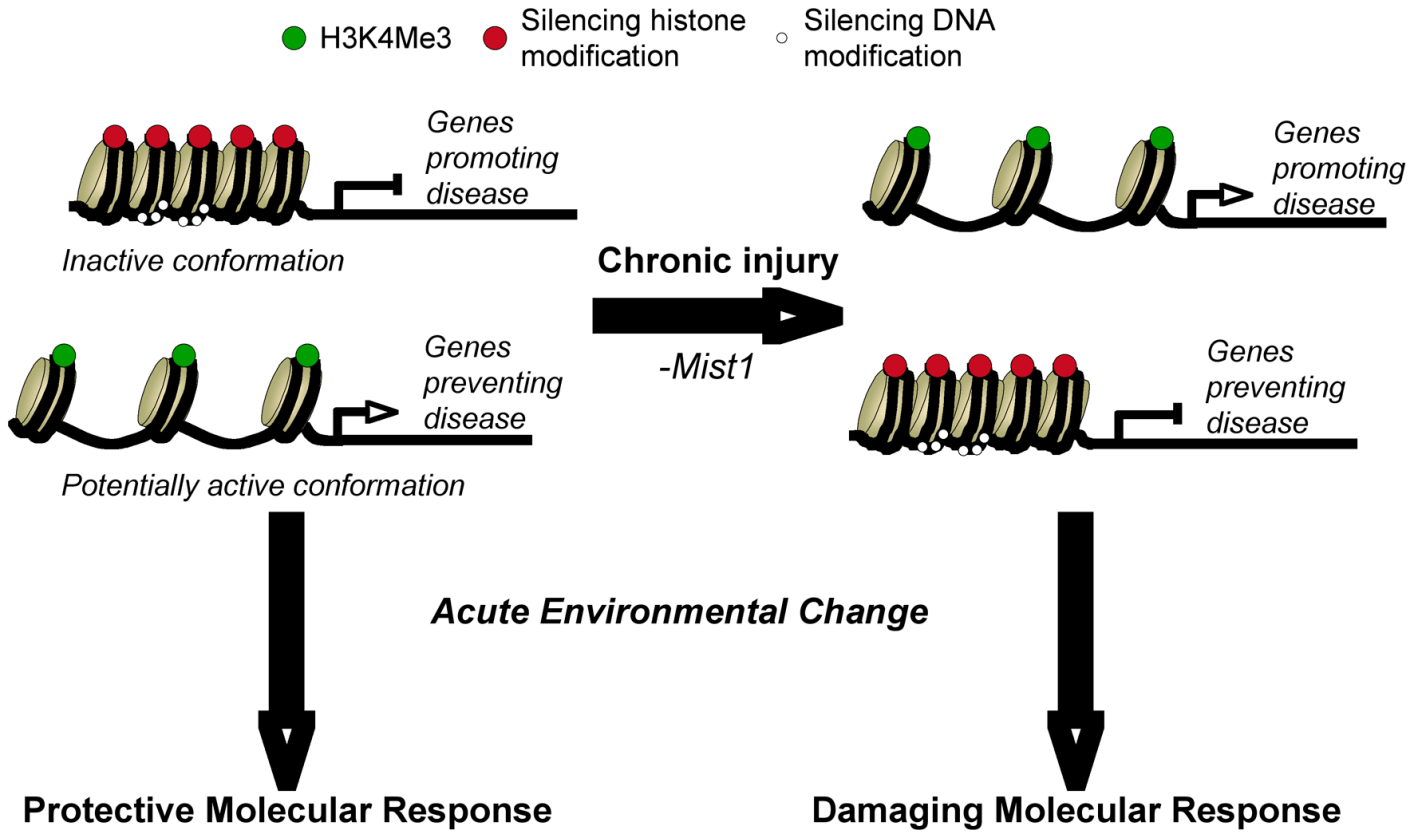
Model for priming of genes for differential expression during CIP. Under normal conditions, genes that promote pancreatic disease are silent while those that protect against injury are either active or primed for activity based on H3K4Me3 enrichment. Following exposure to a chronically injured environment (i.e. loss of MIST1), these genes become reprogrammed so that the response to an adverse environmental challenge now leads to the activation of genes promoting disease progression and severity. This model includes potential epigenetic silencing mechanisms to the DNA or histones, which were not investigated in this study.

Primed or bivalently marked genes have both active and repressive marks at the same promoter, indicative of genes that are within a poised state [26], [45], [46]. Numerous studies have identified the rapid activation of genes in acinar cells within minutes of inducing pancreatic injury, suggesting they are primed for activation [47], [48], [49]. Understanding this relationship could allow us to determine how normal and chronically stressed acinar cells differ in their response to acute challenges. We propose a model in which changes in the H3K4Me3 epigenetic program between genotypes reflects priming of genes that ultimately determine the potential molecular response to changes in cellular environment and the susceptibility to disease (**Figure 7**). In this model, different sets of genes are poised for activation, leading to dramatically different outcomes upon exposure to acute environmental changes. We have shown that *Mist1^−/−^* acinar cells respond dramatically different to acute stress, with limited increases in a number of pathways normally activated in WT cells [23]. The altered response is likely responsible for the increased ability of *Mist1^−/−^* acinar cells to undergo acinar-to-duct cell metaplasia [25] and may dictate the increased sensitivity to pancreatic injury observed in *Mist1^−/−^* mice [22], [23]. We now show in this study that a number of genes linked to pancreatitis and PDAC are differentially enriched for H3K4Me3 in *Mist1^−/−^* acini but are not differentially expressed, suggesting that they are differentially poised to be activated. While the number of genes represented in pancreatitis and PDAC is rather small, this may reflect deficiencies in the analysis, and that many genes involved in these diseases have not yet identified through GO terms. For example, *Fgf21* is epigenetically reprogrammed, with complete loss of H3K4Me3 enrichment in *Mist1^−/−^* mice. While not identified through pathway analysis, we have shown that *Fgf21* is rapidly activated by CIP (100-fold within 60 minutes) and protective against CIP [50]. *Fgf21* is not activated in *Mist1^−/−^* mice in response to CIP and contributes to the pathology exhibited by these mice (Johnson et al, submitted).

In conclusion, this is the first genome-wide study in pancreatic acinar cells identifying H3K4Me3 enrichment. This study provides a map of the H3K4Me3 epigenetic program under normal and chronically stressed conditions, and can be used to understand how genetic and environmental events affect the program globally or specifically. Future work should be directed towards understanding the importance of H3K4Me3 enrichment in genomic regions not associated with TSSs of annotated genes and the repertoire of regulatory regions/genes important for pancreatic acinar cells function.

## Supporting Information

Figure S1 **There is less than a 5% difference in the number of H3K4Me3 peaks (black) and associated genes (white) between wild type (WT) and** ***Mist1^−/−^*** **(M1-) samples.** Total numbers are provided. The parameters used for MACS are as follows: -c (sample 1-Wild Type-IP is the input sample of sample 2-Wild Type-H3K4, sample 4-Mist1^−/−^-IP is the input sample of sample 5-Mist1^−/−^-H3K4), –tsize 49, –bw (192 for sample 2-WT-H3K4, 230 for sample 5-M1-H3K4), –gsize mm, –pvalue 1×10^−5^, –wig, –wigextend 300, –space 50, –diag Ture and –mfold 10,30. BGI gene calling 1.0 algorithm, developed by BGI, was used for gene calling.
(TIF)

Figure S2 **The distribution of scores across all chromosomes exhibits low variability between the wild type (blue) and Mist1^−/−^ (red animals for the H3K4Me3 dataset.** The score equals −10*log10 (P value), where the P value is calculated by MACS.
(TIF)

Figure S3 **The distribution of H3K4Me3 marks shows variability across the chromosomes.** The number of H3K4Me3 enrichment sites was normalized to chromosome size.
(TIF)

Figure S4 **Schematic representation of ChIP-seq data showing H3K4Me3 enrichment for (A) genes with decreased (red) or (B) increased expression (green) in** ***Mist1^−/−^*** **(M1-) pancreatic tissue. Scale bars = 20 kb.**
(TIF)

File S1 **Supplemental Information - References for** **Table 2** **.**
(DOC)

Table S1 **Sequences of primers used for RT-PCR and ChIP-PCR Assays.**
(RTF)

Table S2 **Differential H3K4Me3 Enrichment in WT and** ***Mist1^−/−^*** **tissue.**
(RTF)
