# Supplementary figures and images for: Epigenetic Reprogramming in Mist1−/− Mice Predicts the Molecular Response to Cerulein-Induced Pancreatitis
Source: PLoS One. 2014 Jan 21;9(1):e84182. doi: 10.1371/journal.pone.0084182 (PMC3897368; doi:10.1371/journal.pone.0084182)

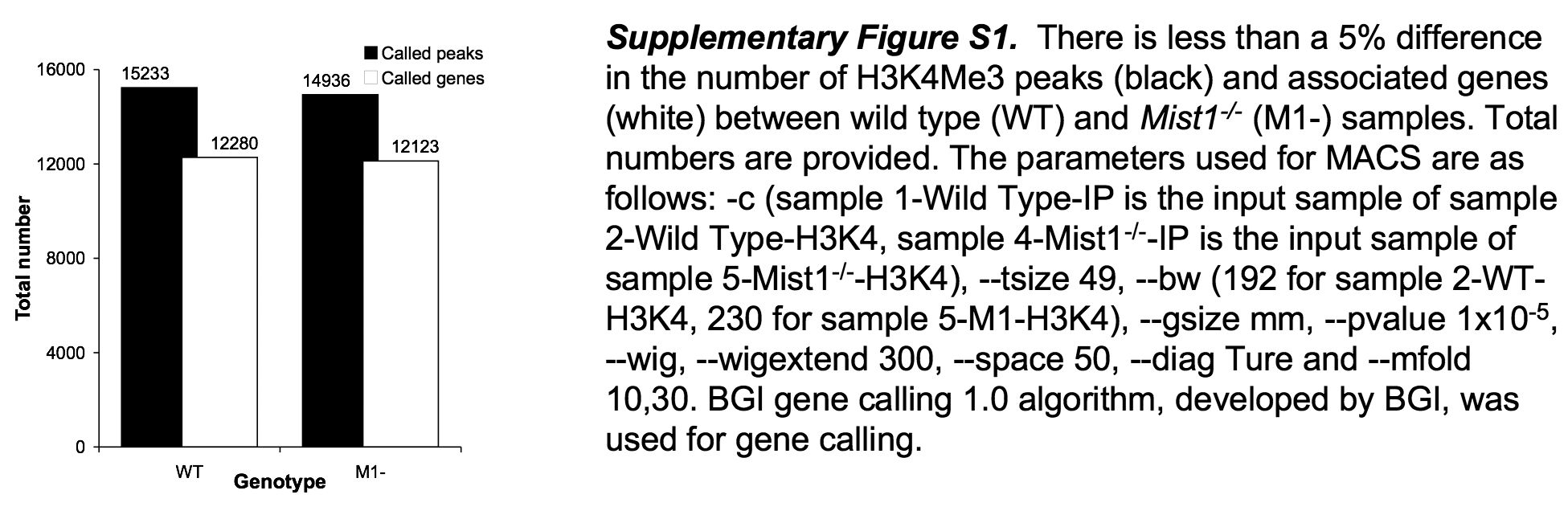

Supplement: Figure S1 — There is less than a 5% difference in the number of H3K4Me3 peaks (black) and associated genes (white) between wild type (WT) and Mist1−/− (M1-) samples. Total numbers are provided. The parameters used for MACS are as follows: -c (sample 1-Wild Type-IP is the input sample of sample 2-Wild Type-H3K4, sample 4-Mist1−/−-IP is the input sample of sample 5-Mist1−/−-H3K4), –tsize 49, –bw (192 for sample 2-WT-H3K4, 230 for sample 5-M1-H3K4), –gsize mm, –pvalue 1×10^−5^, –wig, –wigextend 300, –space 50, –diag Ture and –mfold 10,30. BGI gene calling 1.0 algorithm, developed by BGI, was used for gene calling. (TIF) [file pone.0084182.s001.tif]

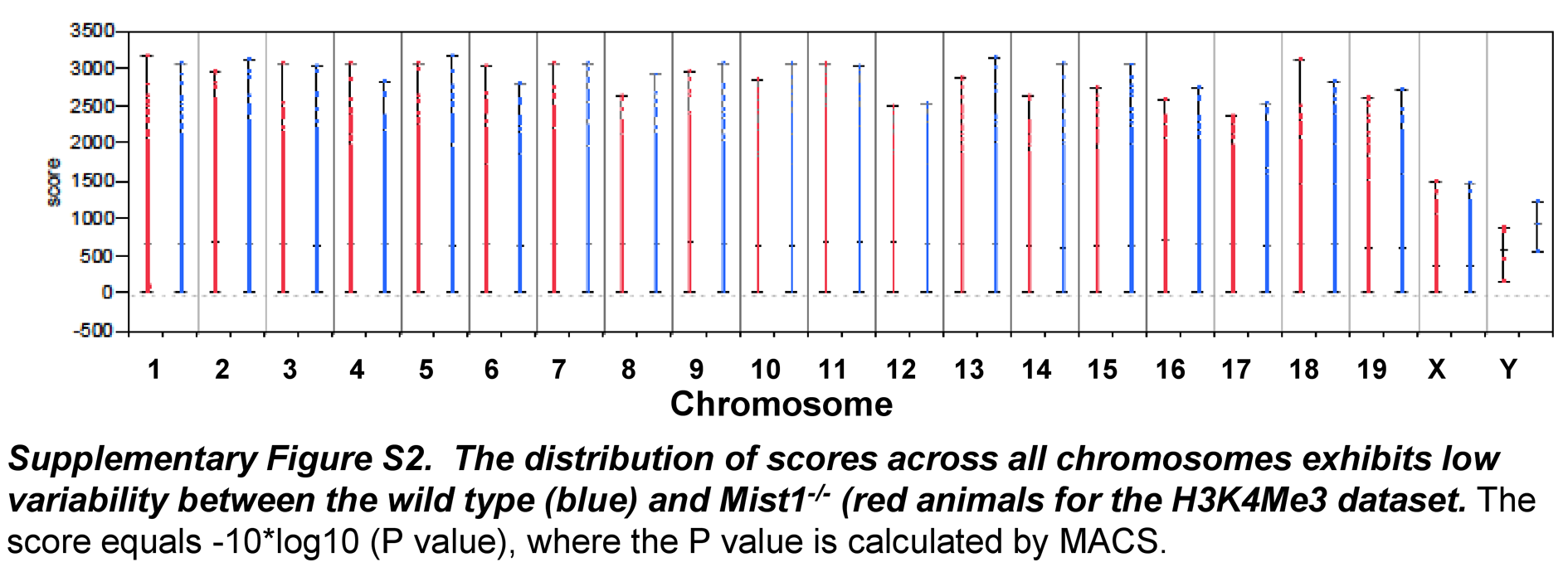

Supplement: Figure S2 — The distribution of scores across all chromosomes exhibits low variability between the wild type (blue) and Mist1−/− (red animals for the H3K4Me3 dataset. The score equals −10*log10 (P value), where the P value is calculated by MACS. (TIF) [file pone.0084182.s002.tif]

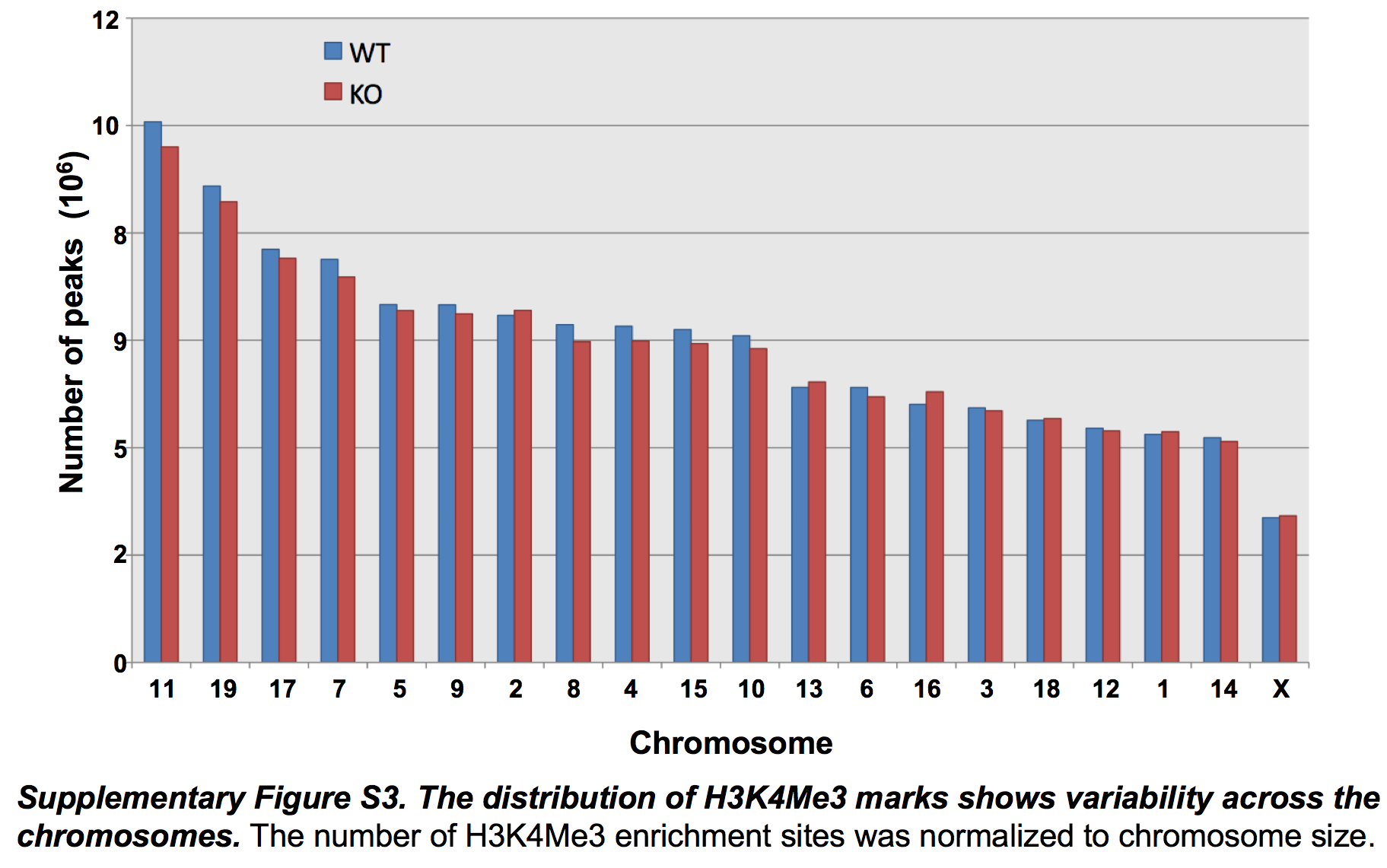

Supplement: Figure S3 — The distribution of H3K4Me3 marks shows variability across the chromosomes. The number of H3K4Me3 enrichment sites was normalized to chromosome size. (TIF) [file pone.0084182.s003.tif]

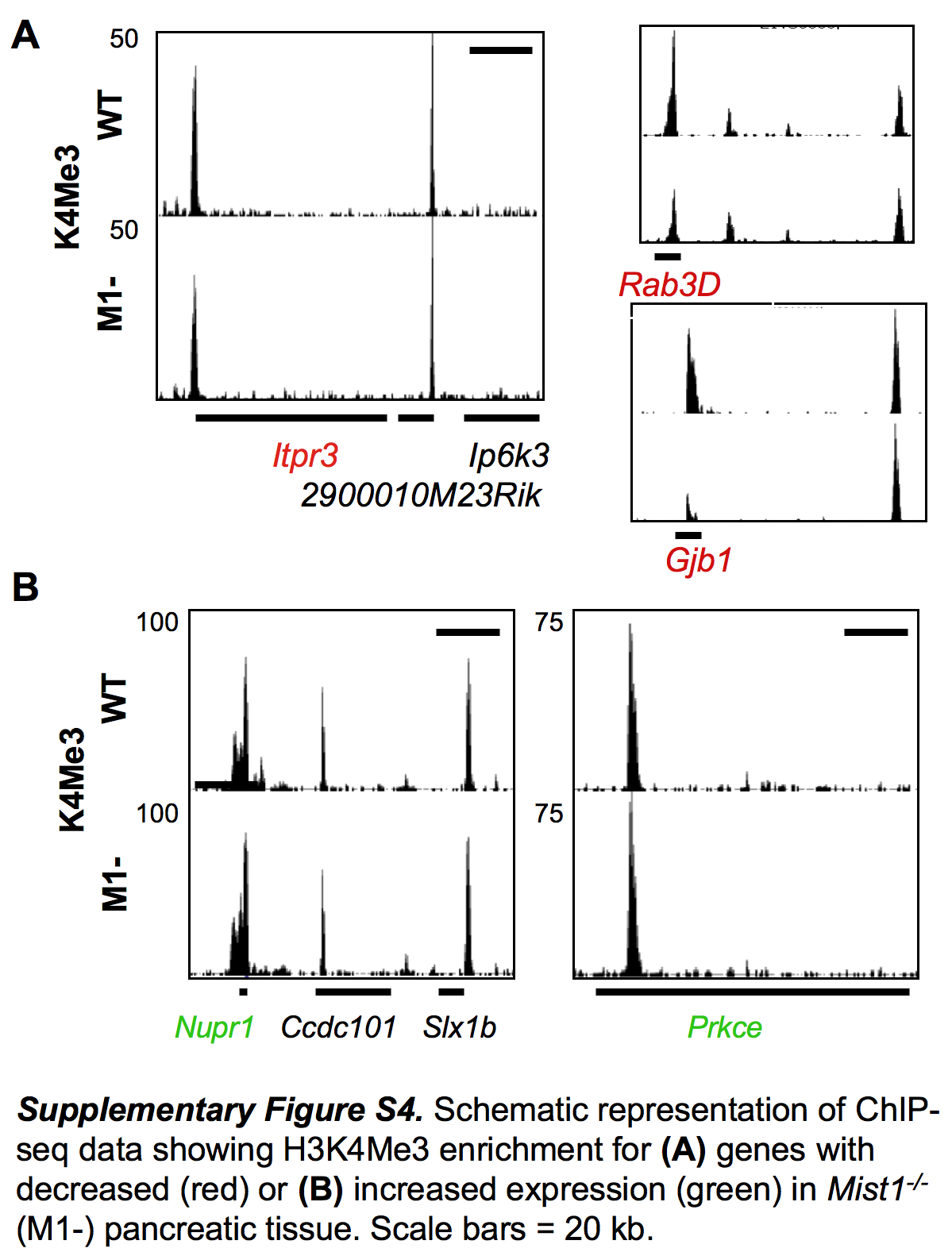

Supplement: Figure S4 — Schematic representation of ChIP-seq data showing H3K4Me3 enrichment for (A) genes with decreased (red) or (B) increased expression (green) in Mist1−/− (M1-) pancreatic tissue. Scale bars = 20 kb. (TIF) [file pone.0084182.s004.tif]
