# Supplementary material for: Epigenetic Reprogramming in Mist1−/− Mice Predicts the Molecular Response to Cerulein-Induced Pancreatitis
Source: PLoS One. 2014 Jan 21;9(1):e84182. doi: 10.1371/journal.pone.0084182 (PMC3897368; doi:10.1371/journal.pone.0084182)
Supplement: File S1 — Supplemental Information - References for Table 2 . (DOC) [file pone.0084182.s005.doc]

***Supplemental Information - References for Table 2***

1. Lohr JM, Faissner R, Koczan D, Bewerunge P, Bassi C, et al. (2010) Autoantibodies against the exocrine pancreas in autoimmune pancreatitis: gene and protein expression profiling and immunoassays identify pancreatic enzymes as a major target of the inflammatory process. Am J Gastroenterol 105: 2060-2071.

2. Jin H, Yu J, Wang W, Chen C, Chen X, et al. (2012) Serum Gc-globulin levels are reduced in patients with severe acute pancreatitis. Eur J Gastroenterol Hepatol 24: 501-505.

3. He S, Wang L, Miao L, Wang T, Du F, et al. (2009) Receptor interacting protein kinase-3 determines cellular necrotic response to TNF-alpha. Cell 137: 1100-1111.

4. Hartwig W, Werner J, Warshaw AL, Antoniu B, Castillo CF, et al. (2004) Membrane-bound ICAM-1 is upregulated by trypsin and contributes to leukocyte migration in acute pancreatitis. Am J Physiol Gastrointest Liver Physiol 287: G1194-1199.

5. Pietruczuk M, Dabrowska MI, Wereszczynska-Siemiatkowska U, Dabrowski A (2006) Alteration of peripheral blood lymphocyte subsets in acute pancreatitis. World J Gastroenterol 12: 5344-5351.

6. Ethridge RT, Chung DH, Slogoff M, Ehlers RA, Hellmich MR, et al. (2002) Cyclooxygenase-2 gene disruption attenuates the severity of acute pancreatitis and pancreatitis-associated lung injury. Gastroenterology 123: 1311-1322.

7. Lugea A, Tischler D, Nguyen J, Gong J, Gukovsky I, et al. (2010) Adaptive Unfolded Protein Response Attenuates Alcohol-Induced Pancreatic Damage. Gastroenterology.

8. Iglesias A, Murga M, Laresgoiti U, Skoudy A, Bernales I, et al. (2004) Diabetes and exocrine pancreatic insufficiency in E2F1/E2F2 double-mutant mice. J Clin Invest 113: 1398-1407.

9. Koh YH, Moochhala S, Bhatia M (2011) The role of neutral endopeptidase in caerulein-induced acute pancreatitis. J Immunol 187: 5429-5439.

10. Hill R, Li Y, Tran LM, Dry S, Calvopina JH, et al. (2012) Cell intrinsic role of COX-2 in pancreatic cancer development. Mol Cancer Ther 11: 2127-2137.

11. Neesse A, Wagner M, Ellenrieder V, Bachem M, Gress TM, et al. (2007) Pancreatic stellate cells potentiate proinvasive effects of SERPINE2 expression in pancreatic cancer xenograft tumors. Pancreatology 7: 380-385.

12. Buchholz M, Biebl A, Neesse A, Wagner M, Iwamura T, et al. (2003) SERPINE2 (protease nexin I) promotes extracellular matrix production and local invasion of pancreatic tumors in vivo. Cancer Res 63: 4945-4951.

13. Weiss FU, Marques IJ, Woltering JM, Vlecken DH, Aghdassi A, et al. (2009) Retinoic acid receptor antagonists inhibit miR-10a expression and block metastatic behavior of pancreatic cancer. Gastroenterology 137: 2136-2145 e2131-2137.

14. Goicoechea SM, Garcia-Mata R, Staub J, Valdivia A, Sharek L, et al. (2013) Palladin promotes invasion of pancreatic cancer cells by enhancing invadopodia formation in cancer-associated fibroblasts. Oncogene.

15. Botta GP, Reichert M, Reginato MJ, Heeg S, Rustgi AK, et al. (2013) ERK2-regulated TIMP1 induces hyperproliferation of K-Ras(G12D)-transformed pancreatic ductal cells. Neoplasia 15: 359-372.

16. Niu GM, Ji Y, Jin DY, Hou J, Lou WH (2010) [Clinical implication of BRSK2 expression in pancreatic ductal adenocarcinoma]. Zhonghua Yi Xue Za Zhi 90: 1084-1088.

17. Kayed H, Kleeff J, Esposito I, Giese T, Keleg S, et al. (2005) Localization of the human hedgehog-interacting protein (Hip) in the normal and diseased pancreas. Mol Carcinog 42: 183-192.

18. Carter H, Samayoa J, Hruban RH, Karchin R (2010) Prioritization of driver mutations in pancreatic cancer using cancer-specific high-throughput annotation of somatic mutations (CHASM). Cancer Biol Ther 10: 582-587.

19. Edling CE, Selvaggi F, Buus R, Maffucci T, Di Sebastiano P, et al. (2010) Key role of phosphoinositide 3-kinase class IB in pancreatic cancer. Clin Cancer Res 16: 4928-4937.

20. Schneider M, Buchler P, Giese N, Giese T, Wilting J, et al. (2006) Role of lymphangiogenesis and lymphangiogenic factors during pancreatic cancer progression and lymphatic spread. Int J Oncol 28: 883-890.

21. Huang X, Ding L, Bennewith KL, Tong RT, Welford SM, et al. (2009) Hypoxia-inducible mir-210 regulates normoxic gene expression involved in tumor initiation. Mol Cell 35: 856-867.

22. Segara D, Biankin AV, Kench JG, Langusch CC, Dawson AC, et al. (2005) Expression of HOXB2, a retinoic acid signaling target in pancreatic cancer and pancreatic intraepithelial neoplasia. Clin Cancer Res 11: 3587-3596.

23. Hagihara A, Miyamoto K, Furuta J, Hiraoka N, Wakazono K, et al. (2004) Identification of 27 5' CpG islands aberrantly methylated and 13 genes silenced in human pancreatic cancers. Oncogene 23: 8705-8710.

24. Hwang RF, Yokoi K, Bucana CD, Tsan R, Killion JJ, et al. (2003) Inhibition of platelet-derived growth factor receptor phosphorylation by STI571 (Gleevec) reduces growth and metastasis of human pancreatic carcinoma in an orthotopic nude mouse model. Clin Cancer Res 9: 6534-6544.

25. Duxbury MS, Ito H, Zinner MJ, Ashley SW, Whang EE (2004) RNA interference targeting the M2 subunit of ribonucleotide reductase enhances pancreatic adenocarcinoma chemosensitivity to gemcitabine. Oncogene 23: 1539-1548.

26. Ruckert F, Joensson P, Saeger HD, Grutzmann R, Pilarsky C (2010) Functional analysis of LOXL2 in pancreatic carcinoma. Int J Colorectal Dis 25: 303-311.

27. Kong B, Michalski CW, Hong X, Valkovskaya N, Rieder S, et al. (2010) AZGP1 is a tumor suppressor in pancreatic cancer inducing mesenchymal-to-epithelial transdifferentiation by inhibiting TGF-beta-mediated ERK signaling. Oncogene 29: 5146-5158.

28. Feldmann G, Habbe N, Dhara S, Bisht S, Alvarez H, et al. (2008) Hedgehog inhibition prolongs survival in a genetically engineered mouse model of pancreatic cancer. Gut 57: 1420-1430.

29. Navas C, Hernandez-Porras I, Schuhmacher AJ, Sibilia M, Guerra C, et al. (2012) EGF receptor signaling is essential for k-ras oncogene-driven pancreatic ductal adenocarcinoma. Cancer Cell 22: 318-330.

30. Bournet B, Pointreau A, Souque A, Oumouhou N, Muscari F, et al. (2012) Gene expression signature of advanced pancreatic ductal adenocarcinoma using low density array on endoscopic ultrasound-guided fine needle aspiration samples. Pancreatology 12: 27-34.

31. Shuno Y, Tsuno NH, Okaji Y, Tsuchiya T, Sakurai D, et al. (2010) Id1/Id3 knockdown inhibits metastatic potential of pancreatic cancer. J Surg Res 161: 76-82.

32. Zhang L, Gao J, Li Z, Gong Y (2012) Neuronal pentraxin II (NPTX2) is frequently down-regulated by promoter hypermethylation in pancreatic cancers. Dig Dis Sci 57: 2608-2614.

33. Sato N, Fukushima N, Hruban RH, Goggins M (2008) CpG island methylation profile of pancreatic intraepithelial neoplasia. Mod Pathol 21: 238-244.

34. Song X, Wang H, Logsdon CD, Rashid A, Fleming JB, et al. (2011) Overexpression of receptor tyrosine kinase Axl promotes tumor cell invasion and survival in pancreatic ductal adenocarcinoma. Cancer 117: 734-743.

35. Song Y, Washington MK, Crawford HC (2010) Loss of FOXA1/2 is essential for the epithelial-to-mesenchymal transition in pancreatic cancer. Cancer Res 70: 2115-2125.

36. Zhang Z, Huang L, Zhao W, Rigas B (2010) Annexin 1 induced by anti-inflammatory drugs binds to NF-kappaB and inhibits its activation: anticancer effects in vitro and in vivo. Cancer Res 70: 2379-2388.
