# Supplementary material for: Epigenetic Reprogramming in Mist1−/− Mice Predicts the Molecular Response to Cerulein-Induced Pancreatitis
Source: PLoS One. 2014 Jan 21;9(1):e84182. doi: 10.1371/journal.pone.0084182 (PMC3897368; doi:10.1371/journal.pone.0084182)
Supplement: Table S1 — Sequences of primers used for RT-PCR and ChIP-PCR Assays. (RTF) [file pone.0084182.s006.rtf]

Supplementary Table S1. Sequences of primers used for RT-PCR and ChIP-PCR Assays
Gene	Gene Region Amplified1	Amplicon Size (bp)	Sequences	Application	
Nptx2 Fw	+648  +811	163	5' ACTGAGGATGCGGGAGAAAT 3'	ChIP-qPCR	
Rv			5' TTTCACCTTTCCCATCGAAC 3'		
Egf  Fw	+493  +631	138	5' GGGACTTGTGCCGGTAAGTA 3'	ChIP-qPCR	
Rv			5' CTTAAGCAAACACGCACACC 3'		
Tm4sf4 Fw	+1020+1160	140	5' CTTCACCCCAGACCGTCTAA 3'	ChIP-qPCR	
Rv			5' AGAACGACTCACCGCAAATC 3'		
Ddc  Fw	+313  +477	164	5' TTGCGTAGTTGGTTGCACAT 3'	ChIP-qPCR	
Rv			5' GGGAATGAAGCGACTAATGC 3'		
Col7a1  Fw	+73 +236	163	5' CCAGCCCAGAGATAGAGGTG 3'	ChIP-qPCR	
Rv			5' CAATTTGGGGAATGCAGAGT 3'		
Nphs1  Fw	+409  +602	193	5' GGGAAAGAAAGACCAGAGGG 3'	ChIP-qPCR	
Rv			5' GATGAATTCGCCTGGGATAA 3'		
Asb11 Fw	   -275 -36	239	5' GGCACCACAGACAGCAGTAA 3'	ChIP-qPCR	
Rv			5' GATTCAATCCAGGAACCCCT 3'		
Sult1c2  Fw	   -202+21	223	5' TAGTGGGGAGTCAGGTGGTC 3'	ChIP-qPCR	
Rv			5' TCTGCAGTCAGTGGGATCAG 3'		
65MB-Chr.7 Fw			5' AAAACAGCCCTTATTTGGGG 3'	ChIP-qPCR	
Rv			5' GTCAGGAGCCTTGTCCTCAG 3'		
80MB-Chr.7 Fw			5' CAGCGGATGCTTTACTGTGA 3'	ChIP-qPCR	
Rv			5' GAAGGGCTGCCAGGTACATA 3'		
Col7a1  Fw			5' GCCCAGAGATAGAGTGACCTG 3'	qRT-PCR	
Rv			5' TCCCAGAGCCAAGTGTATCC 3'		
Nphs1  Fw			5' TCCCCAGAGCATTCAGAGA 3'	qRT-PCR	
Rv			5' AATAAGCAGGTGGAACTCACCTT 3'		
Asb11 Fw			5' TGCATGGAGATTCTGCTGAC 3'	qRT-PCR	
Rv			5' GGTATCCAGCCACTGACCAT 3'		
Sult1c2  Fw			5' CAGGCACCTGGGATGAGTAT 3'	qRT-PCR	
Rv			5' CTGGATTTCATGCTTTGGGT 3'		
Mrpl1  Fw			5' TTGGATATGCCAAGTGACCA 3'	qRT-PCR	
Rv			5' GCTTCTGCCGTTTGAGTTTC 3'		
Ptgs2 Fw			5' AGGACTCTGGTCACGAAGGA 3'	qRT-PCR	
Rv			5' TCATACATTCCCCACGGTTT 3'		
Ripk3 Fw			5' CTGCCTTCCTCTCAGTCCAC 3'	qRT-PCR	
Rv			5' AAGGTTCCTTGTCCGGTAGGG 3'		
Pnliprp1 Fw			5' TCACAGGCCAAGTCAAAGTG 3'	qRT-PCR	
Rv			5' TGGCATCAAACTCATTGGAA 3'		
Axl Fw			5' CCCCCGAGGTACTTATGGAT 3'	qRT-PCR	
Rv			5' CAGATGGTGGAGTGGCTGT 3'		
Hoxb2 Fw			5' CCCCTGGATGAAAGAGAAGA 3'	qRT-PCR	
Rv			5' CAGTTGCGTGTTGGTGTAGG 3'		
Palld Fw			5' TGGCCCAGGAGTACAAAGTC 3'	qRT-PCR	
Rv			5' ACATCTGGATCCTGCACCTC 3'		
Gc Fw			5' CGCCTCTGCCACTTTTAGTT 3'	qRT-PCR	
Rv			5' GCCTTGATTTTTCCTTTCCA 3'		
1 – relative to TSS
