# Supplementary material for: Epigenetic Reprogramming in Mist1−/− Mice Predicts the Molecular Response to Cerulein-Induced Pancreatitis
Source: PLoS One. 2014 Jan 21;9(1):e84182. doi: 10.1371/journal.pone.0084182 (PMC3897368; doi:10.1371/journal.pone.0084182)
Supplement: Table S2 — Differential H3K4Me3 Enrichment in WT and Mist1−/− tissue. (RTF) [file pone.0084182.s007.rtf]

Supplementary Table S2. Differential H3K4Me3 Enrichment in WT and Mist1-/- tissue
Preferential enrichment for H3K4Me3	
1110032F04Rik	Afap1l1	B4galt2 	Ccin  	Cpne9  	
1600016N20Rik 	Afap1l2 	B930041F14Rik	Ccm2l	Crb1  	
1600029O15Rik 	Agpat4 	Bat1a	Cd164l2  	Crlf1  	
1700003E16Rik	agrn	Batf3 	Cd248  	Crlm3	
1700007G11Rik	Agtr1b	BC005764 	Cd300lg  	csf1	
1700013F07Rik	AI646023 	BC028528 	Cd4  	Csf2ra  	
1700023E05Rik 	akap2	bc031353	Cdc25c  	Csgalnact1	
1810010D01Rik	Akap5	bc034090	Cdh13  	Cst6  	
1810041L15Rik	Akr1cl	BC051142 	Cdhr2  	Ctbp2	
2210404O07Rik	Aldh1a1 	BC068157 	cdk1	Cxx1c  	
2610034B18Rik	Alox8	Bdh1	Cdk14  	cyp27a1	
2610203C20Rik 	Angptl2	Bex2 	cdk5rap1	Cyp27b1  	
2700086A05Rik 	Ank1	bhlhe41	Cdkn1c  	Cyp2d22  	
2810459M11Rik 	ank2	Bmp3	Cela2a  	Cyp39a1	
3110082I17Rik	Ankrd34a 	Bmp4 	Cerk  	Cyp46a1	
4632428C04Rik	Ankrd6 	Bmp6 	Cerkl  	D10Ertd610e  	
4930488L21Rik	Ano6	Bnc2 	Ch25h  	D930020B18Rik	
4930544D05Rik	Anxa1	Boc	Chaf1a  	Dab2  	
4930581F22Rik 	anxa6	Btbd17 	Chrna1  	Dach2	
4931423N10Rik 	Apba1	C130071C03Rik 	Chrna7  	Dact2	
5330417C22Rik	apold1	C1qtnf3	Chrne  	Dagla	
9330111N05Rik	Arc	C1rl 	Chst1  	Dbn1  	
9330158H04Rik 	Arhgap25 	C2cd4c 	Chst8  	Dbndd1  	
A030009H04Rik 	Arhgap44	Cabp2	Cldn9  	Dcdc2a  	
A430105I19Rik	Arhgap9 	cacna1c	Clic3  	Dgki	
A930011G23Rik	Arhgef2	Cacna1f 	clk2	Dhh  	
Aass	Arhgef33 	Cacna2d1 	Clmp	Disc1	
Aatk	Arhgef37	Camk2a 	Cnksr2  	Dixdc1	
Abat 	Arhgef39	Car11	Cnnm1	Dlg2	
Abcg1 	Arhgef6 	Car13	Cnr1	Dlgap3  	
Abhd15 	Art5	Car4  	Cnrip1  	Dlk2  	
Acaa1b 	Aspm	Car5b  	Cobl  	Dll3  	
Ace 	atg9b	Cbln1  	Col11a1  	Dll4  	
Acot11	Atoh1 	Ccdc102a  	Col13a1  	Dnahc10  	
Acpp 	Atp13a4 	Ccdc19  	Col27a1  	Dnajc6	
Acsl6	Atp1b2 	ccdc21	Col5a1  	Dnmt3a	
Acy1	Atp2c2	ccdc28b	col7a1	Dock8	
ada	Axl 	Ccdc48  	Colec12  	Dpf1	
Adh1 	B3galt4	ccdc63	coro2b	dpp7	
Adora2a 	B3gat2 	ccdc64	Coro6  	Dse  	
Adora2b 	B3gnt9-ps 	ccdc69	Cpe  	Dusp26	
Adra1d	B4galnt3 	Ccdc74a  	Cpne2  	Dusp8  	
Preferential enrichment for H3K4Me3	
Dym	Fhad1  	Gjc1  	Hdac6	Islr2  	
dync2h1	Fign	Glis1  	Hdgfrp3  	Itpkb	
e2f1	Filip1  	Glp1r	Hepacam  	Iyd  	
Ebi3  	Filip1l	Glt1d1	Hes5  	Izumo4	
Ece2	Fkbp5	Gm10190  	Hfm1  	Jph4	
Ecscr  	Fmnl1  	Gm129	Hist1h2ae  	Kank1	
Ednrb  	Fn3k	Gm14207  	Hist1h2bg  	Kank4  	
Efna3  	Fndc4	Gm2694  	Hist1h2bm  	Kbtbd11  	
Efna4  	Fnip2	Gm7325  	Hk2	Kcna7  	
egf	Foxa1  	Gm88  	Hlf  	kcnab2	
Egln3  	Foxd2	Gm98	Hmga2  	Kcnc3  	
Elavl2  	Foxf2  	Gnat1	Hnf4g  	Kcnh3  	
Elavl4  	Foxp1	Gnat1  	Hoxa1  	Kcnh8  	
Elovl3  	frmd4a	Gnb4  	Hoxa3  	Kcnj4  	
elovl5	Fundc1	Gne	Hoxa4  	Kcnj9  	
Emid1  	Fut1  	Gng8  	Hoxa5  	Kcnk12  	
entpd2	Fxyd3  	Gp5	Hoxb2  	Kcnk6  	
Entpd3	Fxyd5  	Gpc6  	Hoxb3  	Kcnma1  	
Epc1	fxyd6	Gpd1	Hoxb4  	Kcnn2	
Ephb3  	Fxyd7  	Gper  	Hoxc6	Kcp  	
epn3	fzd2	Gpr124  	Hoxd9  	Khdc3	
Erp27	Fzd8	Gpr137b-ps  	Hpdl  	Kif19a  	
Esrrg  	Fzd9  	Gpr30	Hrc  	Kif26a  	
Etnk2  	Galnt2	Gpr44  	Hrk  	Kirrel2  	
Extl1	Galntl2  	Gprc5c	Hs6st3  	Klc3  	
Fa2h  	Galr1  	gpsm1	hsd17b13	Klhl29  	
fam171a1	Galr3  	Gpt	Hspa12a	Klk8  	
Fam189b	gas1	Gpx7  	Hspa12b  	Klk9  	
Fam194a	Gatsl3  	Grap  	Htra1  	Krt7  	
Fam217b	Gbp4  	Grin1  	I133	L3mbtl4  	
Fam43a	Gchfr  	Grrp1  	Icam2  	Lama3  	
Fam46b  	Gck	Gsc  	Id1  	Lats2  	
Fam69b  	Gcnt2	Gucy2g  	Id4	Lcmt1	
Fam70a  	Gdf11  	Gyltl1b	Iffo1  	Lefty1  	
Fbln2  	Gfod1	H2-Q6  	Ifi203  	Lhfp  	
Fbp2  	Gimap1  	Hamp2  	Ifi47  	Lhfpl2	
fbrsl1	Gimap6  	Hapln2  	Il33  	Lipc  	
Fbxl16	Gipc1	Has2  	inha 	Lix1l  	
Fbxl2  	Gipc3  	has2as	Ints5	Lmod1  	
Fbxl7  	Gipr  	Hcn1  	Irf5  	Lmx1a  	
Fes  	Gja1  	Hcn3  	Irx4  	Lonrf3	
Fgf21  	Gjb1	Hcn4  	Isca2	Loxl2  	
Preferential enrichment for H3K4Me3	
Loxl4  	Msx1  	Ntng1  	Plcd1	Rasgrf1  	
Lphn1	Msx1as  	Ntng2  	Pld6  	Rasgrf2  	
Lrfn3  	Msx3  	Nudt17  	Plek	Rassf7	
Lrg1  	Mtap1b	Nup62cl	Pllp	Rcor2  	
Lrrc2  	Mtap6  	Nxph2  	Plxna4  	Rdh12  	
Lrrc26  	Mtmr10	Nxph3  	Pmaip1  	Reep6  	
Lrrc33  	Mtmr12	Oasl2  	Pnliprp2	Relt	
Lrrc4  	Mus81  	obsl1	Pnma1  	Rgag4  	
Lrrc6  	Myct1  	Odf3b  	Pom121l2  	Rgma	
Lrrc9  	Myef2  	Odz1  	Pou3f1	Ric3  	
Lrriq1  	Mylpf  	Olfm2  	Ppfia3	Rimbp3	
Ltb  	Myo7a  	Olfr56  	Ppifos  	Rimkla  	
Ltb4r2  	Nags  	Otud7a  	Ppp1r14c  	Rimklb  	
Ly6a  	Napa	Otx1  	Ppp1r16b  	Ripply3	
Ly6c1  	Ndst1	P2ry2	Ppp1r18	Rnf150  	
Lypd6b  	Nebl  	Padi2  	Ppp1r3d  	Rnf186  	
Mafb  	Nes	Paqr4	Ppp2r5d	Rnf39	
Magix	Neu3  	Pard3	Praf2	Robo4  	
Maml3	Nfasc  	Pcbp4	Prkcb  	Rogdi	
Mamstr  	Nfatc4  	Pcdh12  	Proc  	rp58	
Mapk11  	Nfe2  	Pcdhga10	Prokr1  	Rps6ka2	
March10	Nfe2l3  	Pcsk1	Prom2  	Rragd	
Mctp1	Nfic	Pde10a	prosapip1	Rrm2	
Medag	Nhsl2  	Pde7b	Prr7  	Rtn4r  	
Meis3	Nid2  	Pde9a	Prrg3  	Runx1	
Memo1	Ninl  	Pdgfra  	Prrt4  	S1pr5  	
Mettl24	Nlrx1  	Pdzd4  	Pstpip1  	Saa3  	
Mfsd7a  	Nol3  	Pebp4	Ptgs1  	Samd14  	
Mir125b-1  	Nolc1	Pecam1  	Pth1r  	sardh	
Mir126  	Notch3  	Peg10  	Ptk2b  	Satb1  	
Mir338  	Nphp4	Peg12  	Ptprr  	scamp3	
Mkx	Nphs1  	Peli2  	Ptprt  	Scarf2  	
Mlph  	Npnt  	Phlda2	Pycard  	Scn5a  	
Mme  	Npr3  	phldb1	Pycr1	Scn8a  	
Mndal  	Nptx2  	Phyhipl  	Rab26	Scnn1g  	
Morn3  	Nptxr  	Pigz	Rab6b	Scrib	
Mpl  	Nr1d1	Pik3cd  	Rab9b  	scrn1	
Mreg  	Nr2f1  	Pik3r5  	Radil  	Sec14l2	
Mrgpre  	Nr2f2	Pik3r6  	Raet1a  	Sept1	
Mro	Nrxn2	Pinlyp	ralgps1	Serp2  	
Msh5  	Ntf3  	Plau  	Rapgef3	Sfn  	
Msra	Ntf5  	plcb4	Rapgef5	Sgce  	
Preferential enrichment for H3K4Me3	
Sh2b2  	Snai1  	Tef	Tnfsf9	Utrn	
Sh3kbp1  	Snai3  	Tek  	Tns3	Vash2  	
Shcbp1l	Snca  	Tekt4  	Trak1	Vdr  	
She  	Sncaip  	Tgfb1i1	Trex1  	Vim  	
Shisa7	Sncg  	Tgfb3  	Trim2  	vwa1	
Shoc2	Snx20  	tgfbr3	Trpm3  	Vwce  	
Shox2  	Soga1	Thbs2  	Trpm5  	Vwf  	
Sim1  	Sox17  	Thop1	Tsku	wdr72	
Six1  	Sox18  	Thra	Tspan2	whrn	
Slc1a5	Sox30  	Ticam2  	Ttc22  	Wif1  	
Slc25a41  	Spdef  	Tie2	Ttc34  	Wnt10b  	
Slc25a48	Spdya	Tigd3  	Ttc39c	Wnt3a  	
Slc27a3  	spnb1	Tinag  	Ttc7b	Wnt9a  	
Slc2a13	Srcin1  	Tle3	Ttll9  	Xdh  	
Slc2a5  	Srgn  	Tle4	Tubb2b  	Zc3h12b	
Slc35f1  	Ssc5d	Tll1  	tubg1	Zcchc24	
Slc38a1	St3gal4  	Tm4sf1  	Tubg2  	Zdhhc14	
Slc41a2  	St6galnac3  	Tmc8  	Ube2ql1  	Zfp114  	
Slc4a11  	Stox2	Tmcc2  	Ugt2b34  	zfp238	
Slc4a8  	Sult1a1  	Tmed6  	Ugt8a  	Zfp296  	
Slc52a3	Susd2  	Tmem108  	Unc13a  	zfp334	
Slc6a4  	sv2c	tmem150	Unc45b  	zfp362	
Slc9a3  	Syne4	Tmem191c  	Unc5a  	zfp423	
Slco2a1  	Synpo2  	Tmem210  	Unc79	Zfp831  	
Slco4a1  	sypl2	Tmem229a  	Unc93b1	Zfp872  	
Slfn2  	Syt7  	Tmod2  	Usp31	Zfp932	
Slit3  	Tcfap2a	Tnfsf13b	Usp44	Zpbp  	
Smg7  	Tead1	tnfsf15	Usp54		
					
					
					
					
					
					
					
					
					
					
					
					
					
					
Increased expression in WT	
0610010F05Rik	Apip	Ccrl2	Dixdc1	Fgfr1op2	
1110008F13Rik	Arc	Cdc25a	Dmxl2	Fign	
1110008P14Rik	Arfgap3	Cdc42bpg	Dnajc1	Flt1	
1700019D03Rik	Arfip2	Ceacam1	Dock1	Fn3k	
1810009J06Rik	Arhgef19	Cep44	Dock4	Foxp2	
2010003K11Rik	Asah2	Cep76	Dpf1	Frk	
2410066E13Rik	Asb6	Cgrrf1	Dpp3	Fry	
2610034B18Rik	Asb8	Chuk	Dpp7	Fuom	
2700089E24Rik	Asl	Cib2	Dusp22	Fut1	
4930453N24Rik	Asns	Ckmt1	Dusp26	Fxyd3	
5330417C22Rik	Asun	Cldn8	Dynll1	Fxyd6	
5430419D17Rik	Atp13a1	Cldn9	E230008N13Rik	Gabra4	
6430548M08Rik	Atp13a2	Cln5	Eci3	Gal	
9430008C03Rik	AU040829	Clptm1l	Edem3	Gbgt1	
A130010J15Rik	Aup1	Cmpk2	Eef2k	Gcdh	
Aagab	Azgp1	Cmtm7	Efcab4b	Gck	
Aass	B230307C23Rik	Col7a1	Egf	Gdpd1	
Abcb6	B3galnt2	Copz2	Ehf	Gdpgp1	
Abce1	B3galt4	Cpox	Ehhadh	Gga2	
Abcf1	BC003331	Crb1	Eif2b4	Ggt1	
Abcf2	BC023829	Creb3l2	Eif3j	Gjb1	
Acss1	BC034090	Crlf1	Eif3j	Gjb2	
Adcy9	Bckdhb	Cry1	Eif3m	Glp1r	
Adh1	Bckdk	Csrp1	Eif3m	Gltp	
Agtpbp1	Bhlha15	Ctage5	Eif5a	Gm10334	
Aida	Bicd1	Ctrc	Ell2	Gm10796	
Aig1	Bin1	D10Bwg1379e	Elovl5	Gm15468	
Ak3	Bmp3	D630039A03Rik	Eogt	Gm15470	
Aldh18a1	Brcc3	D930020B18Rik	Ergic1	Gm15698	
Aldh1l2	C1rl	Dagla	Ergic2	Gm6742	
Aldh3a2	C85492	Ddo	Erp27	Gm7278	
Aldh7a1	Cables2	Ddx11	Esrrg	Gmppb	
Alg11	Cabp1	Deaf1	Etf1	Gnb5	
Alg3	Cabp2	Dennd4a	Etnk1	Golga1	
Alg5	Cald1	Depdc6	Extl1	Got1	
Amigo3	Camk2b	Derl3	Faf1	Gpd1	
Ank3	Car4	Dgka	Fam171a1	Gprc5c	
Ankrd24	Ccbl1	Dgkz	Fam212b	Grn	
Anxa6	Ccdc30	Dhcr7	Fam221a	Gsta3	
Aof1	Ccdc56	Dhrs1	Fat3	Gstt3	
Ap3s1	Ccnj	Dhrs7	Fermt2	Gtf2b	
Apba1	Ccpg1	Dio1	Fgf21	Gyltl1b	
Increased expression in WT	
Habp2	Large	Nomo1	Ppapdc1b	Sardh	
Hamp2	Larp4	Npdc1	Ppif	Sec11c	
Hars	Lars	Nphs1	Ppt2	Sec24a	
Hbs1l	Lgals2	Nr4a2	Pqbp1	Sepx1	
Hcfc2	Lmf2	Nucb2	Prdm15	Serf2	
Hdac10	LOC433762	Nudt22	Prdm5	Serp1	
Hid1	Lrrc8b	Nudt4	Prkaa2	Serpini1	
Hist1h4m	Lypd6	Nup62cl	Prom1	Sf4	
Hs2st1	Lyrm9	Nxph2	Prom2	Sfrs18	
Hsd17b13	Lztr1	Odc1	Prrg4	Sfxn1	
Hsd3b7	Mansc1	Ola1	Prss3	Sh3bgrl2	
Htra2	Maoa	Os9	Psat1	Shmt2	
Id2	Map3k13	Osbpl2	Psen2	Siah2	
Idh2	Mctp2	Ostm1	Ptpn21	Sidt2	
Iffo2	Meis3	Otud3	Pwwp2b	Six4	
Ifrd2	Mett11d1	P2rx1	Pycr1	Slc12a2	
Ift20	Mettl7a1	P2rx4	Pycr2	Slc16a5	
Impdh1	Mib1	P2ry14	Qpctl	Slc17a5	
Inpp5b	Mkx	Padi1	Qser1	Slc17a9	
Ints10	Mlph	Padi2	Qsox2	Slc22a26	
Irak1	Mogs	Paip2b	Qtrt1	Slc24a3	
Isca2	Mosc2	Pam	R3hcc1	Slc25a22	
Itga1	Mphosph10	Pbdc1	Rab26	Slc26a6	
Itih4	Mro	Pcbp4	Rab27a	Slc30a1	
Itpka	Mthfd2	Pck2	Rab3d	Slc30a7	
Izumo1	Mtmr10	Pcmtd1	Rab4a	Slc31a2	
Izumo4	Mtmr12	Pdlim1	Ralbp1	Slc33a1	
Jtb	Mtmr6	Pecr	Ranbp3l	Slc35c2	
Kars	Myo15b	Pfas	Rapgef5	Slc35e3	
Kat2a	Myo16	Pi4k2b	Rcl1	Slc35f5	
Kcmf1	Myo1d	Pip5k1b	Rexo2	Slc46a3	
Kcnj14	Myo5c	Pkp4	Rfng	Slc6a4	
Kcnk1	Nans	Pla2g1b	Rhoq	Slc6a9	
Kcnk6	Nbas	Plcd1	Rimbp3	Slc7a1	
Kdelr3	Nckap5	Plcxd2	Riok1	Slc7a4	
Kif16b	Ndst1	Plekha1	Riok3	Slc9a3r2	
Kif1b	Necap1	Plxna2	Rnf160	Slc9a9	
Klf10	Nek6	Polr2e	Rps27l	Slco3a1	
Klhdc7a	Nes	Pomgnt1	Rps6ka2	Smap2	
Klk1b11	Nfasc	Pomt2	Rps6kb1	Smarcc2	
Klk1b3	Nipal2	Pon3	Rragd	Smim14	
Klk1b4	Nnt	Porcn	Rrm2	Smpdl3a	
Increased expression in WT	
Sox7	Tbc1d9b	Tor2a	Tubg2	Whsc1l1	
Spast	Tcf4	Tpd52l1	Uaca	Wwc1	
Spcs1	Tcra	Trabd	Uba5	Xpot	
Spcs3	Tead2	Tram1	Uchl5	Yipf6	
Spdef	Tex30	Trip11	Uck2	Zc3h12b	
Spnb3	Thop1	Trit1	Ufm1	Zdhhc14	
Srgap3	Tia1	Trp53inp2	Ugt2b34	Zfp101	
Srm	Tln2	Try4	Unc13b	Zfp238	
Srp68	Tm6sf2	Tshz2	Unc5a	Zfp280b	
St7l	Tmed6	Tspan1	Unc79	Zfp791	
Stard7	Tmem120b	Tspan13	Upb1	Zfp846	
Susd2	Tmem150	Tst	Upf2	Zfp932	
Syne4	Tmem184c	Tstd3	Usf1	Znhit3	
Sypl2	Tmem214	Ttc13	Vaultrc5	Zranb3	
Syt3	Tmem219	Ttc7b	Wdr59	Zswim1	
Taf9b	Tmem63b	Ttyh1	Wdyhv1	Zyg11a	
Tbc1d30	Tmem9	Tuba4a	 	 	
					
					
Increased H3K4Me3 Enrichment and Expression	
2610034B18Rik	Dixdc1	Gjb1	Nes	Slc6a4	
5330417C22Rik	Dpf1	Glp1r	Nfasc	Spdef	
Aass	Dpp7	Gpd1	Nphs1	Susd2	
Adh1	Dusp26	Gprc5c	Nup62cl	Syne4	
Anxa6	Egf	Gyltl1b	Nxph2	Sypl2	
Apba1	Elovl5	Hamp2	Padi2	Thop1	
Arc	Erp27	Hsd17b13	Pcbp4	Tmed6	
B3galt4	Esrrg	Isca2	Plcd1	Tmem150	
BC034090	Extl1	Izumo4	Prom2	Tstd3	
Bmp3	Fam171a1	Kcnk6	Pycr1	Ttc7b	
C1rl	Fgf21	Meis3	Rab26	Tubg2	
Cabp2	Fign	Mkx	Rapgef5	Ugt2b34	
Cldn9	Fn3k	Mlph	Rimbp3	Unc5a	
Col7a1	Fut1	Mro	Rps6ka2	Unc79	
Crb1	Fxyd3	Mtmr10	Rragd	Zc3h12b	
Crlf1	Fxyd6	Mtmr12	Rrm2	Zdhhc14	
D930020B18Rik	Gabra4	Ndst1	Sardh	Zfp238	
Dagla	Gck				


Preferential enrichment for H3K4Me3	
1110017D15Rik  	Agtr1a  	Brsk2	Crp  	Epha3	
1500009L16Rik  	AI747699  	Bst1  	Cstad  	Epha5  	
1600029D21Rik  	Aip	Bzrap1  	Cuedc1	Ephx2	
1700011H14Rik  	Akap9	C2cd4d  	Cux2  	Epor  	
1700011L22Rik  	Aldh18a1	Cacna1h	Cxx1a  	Eps8R1	
1700030C10Rik  	Aldh3b1	cacnb2	Cxx1b  	Epsti1  	
1700109F18Rik  	Alox5  	cacnb4	Cyp2d22	Es22  	
2010002M12Rik  	Amy2a2  	Calca  	Cyp4f39  	Evc  	
2010015L04Rik  	Amy2a3  	Capsl  	Cytip  	Evpl  	
2410089E03Rik	Amy2a4  	Car3  	D14Ertd668e  	Exoc3l4	
2410137F16Rik  	Amy2a5  	car9	D16Ertd472e	Fam113b	
2810055G20Rik	ank2	Caskin1  	dab2ip	Fam169a	
3110070M22Rik  	Ankrd22	ccbl2	Dak	Fam169b	
3425401B19Rik	Anxa13  	Ccdc11  	dbndd2	Fam171b  	
4833403I15Rik  	Anxa2	ccdc146	Dbp	Fam189a1	
4930422G04Rik	Aox1  	Ccdc158  	Ddc	Fam198a  	
4930452B06Rik  	Ap3m2  	Ccdc37  	Ddit4l  	Fam198b  	
4930550C14Rik  	Apob48r  	Ccdc65  	Dhrs9  	fam81a	
5730559c18rik	App	ccdc76	Diap1	Fam83e  	
6430531B16Rik  	Aqp1  	Ccdc88a  	Dlgap1	Far2  	
7530420F21Rik  	arhgap42	Ccdc89  	Dnaaf3	Faxc	
9130017N09Rik  	Arid5b	Cckbr  	Dnahc11  	Fbn1  	
9530026P05Rik	Arl3	Cct6b	Dnahc6  	Fcho1  	
A230056P14Rik	Armc3  	Cd274	Dnahc7b  	Fetub  	
A930007I19Rik	Armcx2	Cd38  	Dnase2b	Fgf12  	
abca4	Arsg	cd3e	Dpf3	Fgf15  	
Abca8a  	Asb11  	cdc14a	Drd3  	Fgf17  	
Abcc2  	Atg10	cenpl	Dtna	Fgfr1op	
Abcc3  	Atoh7  	Cenpq	Dtwd2	Fgfr4  	
Abcd2  	Atp10d	Cftr  	Duox2  	Fgl1	
Abcg5  	Atp2c1	Chd9	Duoxa2  	Fhl2  	
Ablim1	AY512931  	Chrm1  	Efcab5  	Flnc  	
abParts	b3galnt1	Cisd3  	Efemp1  	Flt4  	
Acbd7  	B3gnt8  	Ckm  	Efemp2  	Fndc5  	
actn1	b4galt5	Cldn6	Eid2	Foxp1	
Adam5  	BC016579	cldn7	Emb  	Fry	
Adam8  	BC037703  	Clstn3  	enho	Frzb  	
Adamts16  	bcar3	Cnksr3	Enpep  	Fut2  	
Adamts20  	Bco2  	Col18a1  	Enpp2  	Fxyd1  	
adamts9	Best1	Col4a3  	Entpd7	Fyb  	
Adcy3  	Bex4  	cops7b	Epb4.1l3  	Gabbr1	
Afm  	Bmp2  	Cpeb3	Epdr1  	Galntl4  	
Preferential enrichment for H3K4Me3	
Galntl6  	Hhex	Kcnh6  	Mgmt  	Npas3  	
Gbp3  	Hhip  	Kcnip4  	Micall2  	Npcd	
Gc  	Hist1h1a  	Kcnj15  	Mir125a  	Npffr2  	
Gck  	Hist1h2ai  	Kcnk6	Mir150  	Nptxr	
Gde1  	Hist1h2an  	Kcnmb2  	Mir17	Nr1h4	
Gid8	Hist1h2bl  	Kcnn4  	Mir17hg	Nr1i2  	
Gja4  	Hist1h2bp  	Kctd1	Mir18  	Nrxn2  	
Glrx	Hist1h3g  	Kctd19  	Mir19a  	Nrxn3  	
Gm10451  	Hist1h4f  	Khdrbs3  	Mir19b-1  	NSR1	
Gm14164  	Hivep2	Kif12  	Mir20a  	Ntrk2  	
Gm15800	Hmcn1  	kifap3	Mir210  	Numbl  	
Gm1673  	Hmgcs2	Klf14  	Mir26b  	Nup62cl	
Gm347  	Hmgn3  	Klf8	Mir761  	Nxf2  	
Gm4349  	Hormad1  	Klhdc9  	Mir92-1	Nyap1	
Gm4535  	Hoxd3  	Krt79  	Mir99a	odz2	
Gm5801  	Hrasls5  	Krt80  	Mirlet7e  	Olfr1033  	
Gm609  	Hspbp1	L3mbtl  	Mnd1	Onecut1	
Gm6792  	Htr4	Lcat  	Mns1	Osmr	
Gm7120  	I830012O16Rik  	Lgals12  	Mobkl2a	Oxr1	
Gmpr  	Icam1  	Lgals3  	mpz	pak6	
gnas	Ifi44  	Lgals3bp  	Mtap7d3  	palld	
Gng11  	Ifit1  	Lgals9	Mtus2  	Pamr1  	
Gpam	Igdcc3  	Liph  	Myt1l  	Pappa	
Gpc3  	Igfals  	Lpar4  	N28178  	pard3b	
Gpc4	Igsf23 	Lpin3	Naip6  	Pcdh9  	
Gpr22  	Igsf9b  	Lrfn4	Naip7  	Pcdhb1  	
gpr39	Ikzf4  	Lrp2bp  	Nars2	pcp4l1	
Gpx2  	Il13ra1	Lrp3  	Nav2	Pcsk5  	
Grb14	Il18bp  	Lrpprc	Nbl1  	Pcx	
Greb1l	il7	Lrrc18  	Ncam2	Pde1a  	
Gria3  	Immp2l	Lrrc31	Ncmap	Pdgfd	
Gsdmc2	Ing5  	Lrrc36  	Ndufv1	Pdgfr1	
Gsdmc4	Inpp4b  	Lrrc3b  	Nedd4l	Pdlim4  	
gsn	Iqsec2	Lrrc49	Neil2  	Pdlim5	
Gstm7  	Isg20	Mamdc2	nespas	Pgk1	
Gucy1b3	Itfg3	Maml3	Neurl1a  	Pgm2	
Gypc  	Itga2  	map4k4 	Neurl3  	phldb1	
Hdx  	Itga8  	Map6d1  	Ngfrap1	Pi4ka	
Hebp2	Itih5  	Mapk15  	Nkain2  	Piga	
hecw2 	Itm2a  	Mark2	Nostrin  	Pigt	
Heph  	Jub  	Mast4	Nova1	Pik3cg	
Hey1	Kcne3  	Mef2c	Nox4  	Piwil4  	
Preferential enrichment for H3K4Me3	
Plcxd3  	Rnf207  	Slc25a34  	Tbc1d10c  	Vps4a	
pls1	Rprm	Slc26a7  	TBC1d4	Wdfy4  	
Plscr1	Rsad2  	Slc28a3  	Tbcb	Wdr41	
Pm20d1  	Rsph1  	Slc38a4	Tbx1	Wdr63  	
Pml	Rsph4a  	Slc41a1  	tcrb	Wfdc10  	
Pnliprp1	Rufy1	slc4a2	Tesc  	Wfdc15b  	
Podn  	Rufy2	slc4a4	Tex14	Wfdc9  	
Pola2	Rundc3a  	Slc4a9  	Tgfb2  	wipf1	
Ppp1r3a  	Runx2	Slc5a1  	Thbd  	Wnt4  	
Ppp2r2b  	samd9l	Slc5a8  	Timp1  	Xpnpep3	
Praf2  	samhd1	slc7a11	Tinag	Zbtb20	
Prelp  	sass6	Slc8a1	Tinagl1	Zdhhc15  	
Prickle1	Scamp1	Smad9  	Tm4sf4	zfhx4	
Prkar1b  	Scarf2	Smarca2	Tmc5	Zfp119	
Prkce	Scd3  	Smim6	Tmcc3	Zfp131  	
Prkd2	Scn1b	Smtnl1  	Tmem117  	Zfp286  	
Prr5l  	Scrt2  	Snhg3	Tmem136  	Zfp36	
Psmc1  	Sctr  	Sntg2	Tmem150c  	Zfp366  	
Ptger2  	Sema3f  	Snx12	Tmem171  	Zfp397os	
Ptgfr  	Serpinb6b  	Sp7  	Tmem178  	Zfp407	
Ptgs2  	serpinb8	Spag1  	Tnfaip8	Zfp41	
Ptn  	serpine2	Spata24	Tnfrsf19	Prrxl1	
Ptplad2  	Setd4	Srrm2	Treh  	Prss23	
ptprj	Sgk2  	Srrm4  	Trib2	Prune2  	
Pxn	Sgms1	ssbp4	Trim5	psen2	
Rab36  	sh2b2	St8sia6  	Trim50	Scx  	
Rab37  	Sh2d5  	Stab1  	Trim6  	Sdc3  	
Rab42-ps  	Sh3bgrl	Stard6	Trpm6  	Sdsl  	
Rangrf  	Sh3pxd2a	Stat4  	Tshz1	Sec16b  	
Rara	Shank3	stx6	Tsix  	Snx22  	
Rasal3  	Shisa2  	Stxbp5l  	Ttc23	Sorcs1	
Rasl2-9-ps  	Shroom3	Stxbp6	Ttll10  	Sox5  	
Rassf2  	Sipa1l3  	Sucnr1  	Ttr	Sox6	
Rbms2	Sirpa  	sulf2	Tut1	Tmem37  	
Rdh5  	Ska1  	Sult1c2  	Unkl  	Tmem45a  	
Rftn1  	Slain1	Suv420h2	Usp54	Tmem82  	
Rgnef	Slc11a1  	Syn1  	Vav3  	Tmod1  	
Rims2	Slc15a2  	syne1	Veph1  	Zfp521	
Ripk3  	Slc18a2  	syne2	Vmn2r11  	Zfp532  	
rnase1	Slc1a1  	Syt1  	Vmn2r8	Zfp772	
Rnf180  	Slc1a5	Taok3  	Vps13a	Zik1  	
	
Increased expression in Mist1-/-	
0610011F06Rik	Als2	Caprin2	Cnpy4	Efcab4a	
1110008L16Rik	Ammecr1	Car8	Cog5	Efr3b	
1190003J15Rik	Anapc13	Car9	Commd6	Eif2b2	
1600029D21Rik	Ang	Casc4	Commd8	Eif3k	
1700019G17Rik	Angptl1	Casd1	Comt1	Elmo3	
1700040L02Rik	Ankmy2	Casp9	Cope	Emd	
1700112E06Rik	Ankrd22	Cat	Cox17	Eml3	
1810011O10Rik	Ankzf1	Ccbe1	Cox6a1	Emp2	
2310003L22Rik	Ap3s2	Ccdc141	Cox6c	Enho	
2810026P18Rik	Ap4b1	Ccdc167	Cox7a1	Enpp1	
3110002H16Rik	Ap4m1	Ccdc68	Cox8a	Epb4.1l4a	
3110057O12Rik	Apcs	Ccni	Cpne3	Epc2	
8430419L09Rik	Aph1c	Cd24a	Cpt1a	Epcam	
9030624J02Rik	Arap2	Cd320	Cryzl1	Epha7	
9130230L23Rik	Arf3	Cd3e	Cs	Ephx2	
Abcb8	Arl10	Cd59a	Ctbp2	Eps8l3	
Abcg5	Arpp19	Cd59b	Ctps2	Erlin2	
Abhd2	Arsg	Cd81	Cyp4v3	Etfb	
Abhd3	As3mt	Cdc14b	Cyp7b1	Ezr	
Acadl	Asb11	Cdc42ep5	D10Wsu102e	Fads3	
Acadvl	Asrgl1	Cdkl1	D15Ertd621e	Fam13a	
Acat1	Atl3	Cep70	D230025D16Rik	Fam176b	
Acer2	Atp10d	Ces2g	D7Ertd443e	Fam178a	
Acnat1	Atp5h	Cetn2	Dazap2	Fam195b	
Acot13	Atp6v0e	Chd3	Dctn4	Fam214a	
Acsl3	Atp6v1a	Chp1	Ddb2	Fam48a	
Acsl5	Atpif1	Chst15	Ddc	Fam81a	
Adam10	B4galnt1	Cilp	Decr1	Far2	
Adam17	Bat3	Cirbp	Dek	Fbxo21	
Adamts9	BC026585	Ckm	Dhcr24	Fchsd2	
Aff1	Bcl11a	Clca2	Dhdh	Fetub	
Afm	Best1	Cldn10	Dhrs11	Fggy	
Agpat2	Bloc1s3	Cldn18	Dlat	Fh1	
Agr2	Bphl	Cldn6	Dnajc12	Fhl1	
Agtrap	Brca1	Cldn7	Dock7	Fkbp9	
AI462493	Bspry	Clic6	Dtna	Foxo3	
Aifm3	Bst2	Cln6	Dtwd2	Foxp1	
Aip	C1qa	Cmbl	Dtx2	Foxred2	
Akap8l	Cachd1	Cml2	Duox2	Fpgt	
Akip1	Cad	Cml4	Dusp16	Ftsjd2	
Akr1a4	Camta1	Cnot10	Dynll2	Gadd45a	
Increased expression in Mist1-/-	
Aldh1a1	Capns1	Cnot6l	Ech1	Galk1	
Galm	Hsdl2	Manba	Nipsnap3a	Pgcp	
Garnl4	Hsp90aa1	Map4k4	Nit1	Phf16	
Gata4	Hyal3	Mapk13	Nmt2	Phpt1	
Gbas	Hypk	Marcks	Notch1	Phyh	
Gbe1	Ifi27l1	Mboat7	Nox4	Piga	
Gc	Ifi30	Mcmbp	Noxa1	Pigb	
Gda	Ift52	Me3	Nr1h4	Pigyl	
Gde1	Igbp1	Mecom	Nr2c2	Pik3ap1	
Gdf10	Igdcc4	Med10	Nradd	Pip5k1a	
Gfra1	Igfals	Mepce	Nrp1	Pipox	
Gif	Ilf3	Mia1	Nt5c3l	Pitpnc1	
Glod4	Immt	Mid1ip1	Ntn1	Pla2g4a	
Glul	Impa1	Midn	Ntn4	Pla2g5	
Gm11818	Inpp5e	Mier1	Nub1	Plekhb1	
Gm12528	Ints12	Mmp15	Nudc	Plin3	
Gng5	Isyna1	Mocs2	Nudt14	Pls1	
Gnl3l	Itfg3	Moxd1	Ocln	Pls3	
Golga7	Itgbl1	Mpv17l	Ocrl	Plxnb2	
Gpc1	Itm2a	Mr1	Ogdh	Pm20d1	
Gpi1	Itsn1	Mrpl23	Olfr1029	Pnck	
Grcc10	Ivns1abp	Mrpl52	Oxct1	Pnpo	
Gria3	Jak2	Mrrf	Pafah1b3	Polr1a	
Gstk1	Kcnj15	Mta1	Paip2	Por	
Gstm1	Kctd14	Mta3	Pak6	Ppap2b	
Gstm2	Kdm3a	Myo1b	Pank1	Ppme1	
Gstm3	Kel	Naaladl2	Paqr5	Ppp1r1b	
Gstm4	Klf12	Naip1	Parm1	Prdx2	
Gstm5	Klhdc6	Naip5	Parp11	Prdx3	
Gstm7	Klhl11	Naip6	Pawr	Prelid2	
Gsto1	Klhl13	Nalcn	Pcmtd2	Prkag1	
Gtf3c1	Krt20	Naprt1	Pcolce	Prkd3	
Gtl3	Lama5	Nat12	Pcp4l1	Prpf6	
Gtpbp10	Lass4	Nat5	Pde8b	Prpsap2	
Gys1	Lcat	Nbl1	Pdha1	Psma6	
H2afv	Ldha	Ndrg2	Pdik1l	Psmb6	
Hadh	Ldhb	Ndufa7	Pdk1	Psme1	
Hadha	Leprotl1	Ndufaf5	Pebp1	Psme2	
Heatr7a	Liph	Ndufs4	Peg3	Ptch1	
Heph	Lmo4	Ndufs8	Pex6	Ptdss1	
Hmgcs2	Lsm6	Nfatc2ip	Pfkl	Ptgr1	
Hmox2	Mak10	Nfe2l2	Pga5	Ptn	
Increased expression in Mist1-/-	
Hpgd	Man2c1	Ngfrap1	Pgap2	Ptprj	
Pts	Slc18a2	Stard13	Tmem195	Unc119	
Pvrl2	Slc22a1	Stat5a	Tmem205	Uqcr10	
Rab11b	Slc25a1	Stbd1	Tmem209	Uqcrb	
Rab11fip4	Slc25a4	Stk16	Tmem231	Urod	
Rab25	Slc26a7	Suclg2	Tmem30b	Use1	
Rabl3	Slc26a9	Sucnr1	Tmem41b	Usp2	
Rad21	Slc30a2	Sulf2	Tmem47	Usp40	
Rcbtb2	Slc35d1	Sult1c2	Tmem50b	Uxt	
Rce1	Slc37a4	Surf1	Tmem85	Vegfb	
Rest	Slc39a8	Swi5	Tmem86a	Vps11	
Rgl2	Slc40a1	Syt15	Tmprss2	Vps28	
Rnd2	Slc44a3	Taf1a	Tmx4	Vps53	
Rnf114	Slc7a6	Tapbp	Tnfaip8	Vsig2	
Rnf125	Slc9a8	Tapt1	Tnfsf10	Vstm4	
Rnf167	Smagp	Tbc1d17	Tnip1	Wars	
Rnft1	Smarca1	Tbc1d22a	Tox	Wdr19	
Rod1	Smim20	Tbc1d24	Tpk1	Wdr51b	
Rorc	Smim3	Tbccd1	Tpp2	Wdr67	
Rprm	Smpd2	Tdh	Trappc10	Wdr72	
Rrm2b	Snap29	Tecr	Trim41	Wfdc10	
S100a1	Snapin	Tek	Trip6	Wfdc9	
Sbds	Snrpd2	Tes	Trove2	Wtip	
Scaper	Snx1	Thra	Trrap	Wwp1	
Scrn3	Snx5	Tifa	Tspan12	Zbtb37	
Scx	Snx6	Tk2	Tspyl2	Zc3h12c	
Sdha	Soat1	Tkt	Ttc15	Zfp113	
Sdhd	Socs2	Tlcd1	Ttc25	Zfp229	
Sdsl	Sod1	Tle1	Ttll6	Zfp266	
Sema7a	Sord	Tle6	Tuft1	Zfp426	
Serpina6	Sphk1	Tm4sf4	Tulp2	Zfp654	
Sfrp1	Spire1	Tmc4	Txndc12	Zfp827	
Sh3bgrl3	Spire2	Tmc7	Txndc14	Zkscan5	
Sh3pxd2a	St6gal1	Tmem116	Txndc14	Zmynd8	
Sirt4	St6galnac2	Tmem19	Ube2i	Zp3	
Skiv2l2	Stambpl1	 	 	 	
					
					
					
					
					
					
Increased H3K4Me3 Enrichment and Expression	
1600029D21Rik	Cldn6	Gc	Naip6	Scx	
Abcg5	Cldn7	Gde1	Nbl1	Sdsl	
Adamts9	D15Ertd621e	Gria3	Ngfrap1	Sh3pxd2a	
Afm	Ddc	Gstm7	Nox4	Slc18a2	
Aip	Dtna	Heph	Nr1h4	Slc26a7	
Ankrd22	Dtwd2	Hmgcs2	Pak6	Sucnr1	
Arsg	Duox2	Igfals	Pcp4l1	Sulf2	
Asb11	Enho	Itfg3	Piga	Sult1c2	
Atp10d	Ephx2	Itm2a	Pls1	Tm4sf4	
Best1	Fam81a	Kcnj15	Pm20d1	Tnfaip8	
Car9	Far2	Lcat	Ptn	Wfdc10	
Cd3e	Fetub	Liph	Ptprj	Wfdc9	
Ckm	Foxp1	Map4k4	Rprm		
